# Optimal Electrocatalyst Design Strategies for Acidic Oxygen Evolution

**DOI:** 10.1002/advs.202401975

**Published:** 2024-08-09

**Authors:** Dongdong Zhang, Qilong Wu, Liyun Wu, Lina Cheng, Keke Huang, Jun Chen, Xiangdong Yao

**Affiliations:** ^1^ State Key Laboratory of Inorganic Synthesis and Preparative Chemistry College of Chemistry Jilin University Changchun 130012 P. R. China; ^2^ Intelligent Polymer Research Institute and ARC Centre of Excellence for Electromaterials Science Australian Institute for Innovative Materials University of Wollongong Wollongong NSW 2500 Australia; ^3^ Institute for Green Chemistry and Molecular Engineering Sun Yat‐Sen University Guangzhou Guangdong 510275 P. R. China; ^4^ School of Advanced Energy and IGCME Shenzhen Campus Sun Yat‐Sen University (SYSU) Shenzhen Guangdong 518100 P. R. China

**Keywords:** acidic OER, catalyst design, defect engineering, phase engineering, structure engineering

## Abstract

Hydrogen, a clean resource with high energy density, is one of the most promising alternatives to fossil. Proton exchange membrane water electrolyzers are beneficial for hydrogen production because of their high current density, facile operation, and high gas purity. However, the large‐scale application of electrochemical water splitting to acidic electrolytes is severely limited by the sluggish kinetics of the anodic reaction and the inadequate development of corrosion‐ and highly oxidation‐resistant anode catalysts. Therefore, anode catalysts with excellent performance and long‐term durability must be developed for anodic oxygen evolution reactions (OER) in acidic media. This review comprehensively outlines three commonly employed strategies, namely, defect, phase, and structure engineering, to address the challenges within the acidic OER, while also identifying their existing limitations. Accordingly, the correlation between material design strategies and catalytic performance is discussed in terms of their contribution to high activity and long‐term stability. In addition, various nanostructures that can effectively enhance the catalyst performance at the mesoscale are summarized from the perspective of engineering technology, thus providing suitable strategies for catalyst design that satisfy industrial requirements. Finally, the challenges and future outlook in the area of acidic OER are presented.

## Graphical abstract

This review systematically summarizes, classifies, and discusses advanced acidic OER catalysts in recent years according to three design strategies (defect, phase, and structure engineering). The basic research works on enhancing the performance of catalysts are emphasized, providing atomic‐level insights for acidic OER catalysis. Furthermore, this review also summarizes the challenges and prospects of catalysts, offering guidance for future developments.

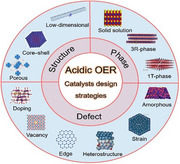

## 1 Introduction

In recent years, owing to the over‐exploitation and consumption of fossil resources, the resulting greenhouse effect has severely damaged the ecological environment through global warming, rising sea levels, and land desertification; this has received widespread attention, considering the rapid development of various industries.^[^ ^1^ ^]^ The increasing CO_2_ concentration in the atmosphere necessitates the development of clean energy resources that can suitably replace fossil resources, thereby solving these environmental problems and sustaining human society. Hydrogen has emerged as the most promising candidate to satisfy future energy requirements because of its high energy density and environmental friendliness; consequently, it has attracted increasing attention.^[^ ^2^ ^]^ Hydrogen is industrially produced through separation and purification from water gas and petroleum cracking, which are associated with fossil energy production. Currently, ≈96% of hydrogen is produced from fossil fuel in this manner for large‐scale utilization.^[^ ^3^ ^]^ Notably, conventional fossil fuels continue to be used. Furthermore, the yield and selectivity of the obtained hydrogen are unsatisfactory, indicating that additional cost and time would be spent on optimizing the system. Theoretically, the multi‐iteration of technology is necessary for this process; however, its cost is difficult to estimate. Electrochemical water splitting (EWS), which differs from conventional methods in the chemical industry, has emerged as a viable approach to obtain high‐purity hydrogen on a large scale. EWS can be powered by renewable energy sources such as wind, tidal, and solar energy and has been widely applied to the large‐scale industrial production of hydrogen.^[^ ^4^ ^]^

EWS systems are conventionally categorized into acidic, neutral, or alkaline configurations based on the pH of the electrolyte.^[^ ^5^ ^]^ In contrast to neutral and alkaline EWS systems, the acidic proton exchange membrane water electrolyzer (PEMWE; an acidic EWS system) is more favorable for industrial operations because it provides rapid proton transfer, increased conductivity, and decreased ohmic resistance. These attributes enable the acidic PEMWE to achieve high current densities. Additionally, the hydrogen produced by the PEMWE effectively mitigates gas crossover concerns attributed to the operation of the system at high pressures; therefore, pure hydrogen can be obtained.^[^ ^6^ ^]^ EWS involves two distinct half‐reactions: the hydrogen evolution reaction (HER) at the cathode and the oxygen evolution reaction (OER) at the anode.^[^ ^7^ ^]^ In detail, the dominant OER mechanism in acidic electrolyze mainly includes the adsorbate evolution mechanism (AEM) and lattice oxygen mechanism (LOM). First, the common steps of AEM and LOM, the adsorption H_2_O is translated to O* after twice deprotonations on the surface of catalysts.

(1) $$H_{2}O+*\to \mathrm{HO}*+H^{+}+e^{-}$$

(2) $$\mathrm{HO}*\to O*+H^{+}+e^{-}$$

In AEM, another H_2_O molecule reaction with O*, causing the formation of O‐O bonds and HOO* intermediate. Finally, the oxygen molecule is generated after deprotonation and released from the active sites.

(3) $$O*+H_{2}O\to \mathrm{HOO}*+H^{+}+e^{-}$$

(4) $$\mathrm{HOO}*\to O_{2}+H^{+}+e^{-}$$

In LOM, O* coupling with a lattice O atom to form an oxygen molecule and an oxygen vacancy (V_O_). The formed V_O_ filled with O atom from another H_2_O molecule after the third deprotonation and formed adsorbed hydrogen. Finally, the adsorbed hydrogen is released after the fourth deprotonation.

(5) $$O*+O_{L}\to O_{2}+V_{O}$$

(6) $$V_{O}+H_{2}O\to O_{L}+H*+H^{+}+e^{-}$$

(7) $$H*\to *+H^{+}+e^{-}$$

Notably, the four‐electron transfer involved in the OER at the anode (2H_2_O → O_2_ + 4H^+^ + 4e^−^) encounters a considerably higher energy barrier than that for the two‐electron transfer in the HER at the cathode (2H^+^ + 2e^−^ → H_2_). This difference in energy requirements highlights the pivotal role of the OER in the EWS mechanism.^[^ ^3^, ^8^ ^]^ The OER necessitates a higher external potential to facilitate the reaction. Consequently, the development of highly active catalysts is imperative to minimize the energy barrier of the reaction. Particularly, the anodic segment encounters considerable challenges, including high oxidation overpotential and the creation of localized acidic environments during proton transport, which hinder the formation of optimal active sites. Extensive studies have demonstrated that materials based on Ir and Ru represent state‐of‐the‐art catalysts for the acidic OER.^[^ ^9^ ^]^ The cost of commercial PEMWE catalysts have been a challenge to meet the 2025 goal of the US Department of Energy (US DOE H_2_ production cost <2 USD per 1 kg H_2_).^[^ ^10^ ^]^ Besides, the cost for Ir (US $60670 kg^−1^) is much more expensive than Ru (US $9523 kg^−1^), which accelerates the application of Ru in PEMWEs in the future.^[^ ^11^ ^]^ However, the current density standard of 3 A cm^−2^@1.9V of the US DOE is also a significant challenge to achieve for Ru‐based materials.^[^ ^12^ ^]^ Therefore, the development of advanced and low‐cost acidic OER catalysts is the key to sustainable PEMWEs. Ir‐based materials are mainstream catalysts for PEMWE, owing to their long‐term stability in acidic media. However, the activity of Ir‐based materials is still a challenge and should be further enhanced to reduce the meaningless consumption of energy. Besides, the low Ir content in the Earth's crust limits the large‐scale application of these materials.^[^ ^13^ ^]^ The development of low‐Ir‐content catalysts, even isolated Ir atom catalysts, has long been the focus of acidic OER research. Especially, how to maintain or even improve the activity and stability under the premise of reducing the amount of Ir to maximize the atomic utilization and meet the economic effect is a major challenge for Ir‐based materials. Finding elements with high crustal abundance and meeting the requirements of acidic OER to replace Ir has become an important research branch.

Studies have revealed that Ru‐based materials and some transition metal‐based materials can be excellent substitutes for Ir‐based materials owing to their suitable electronic structures and more abundant storage capacities. Ru‐based catalysts are relatively abundant in Ir and demonstrate high activity, particularly in the context of the acidic OER. The adjustability of their electronic structure enables favorable binding energies with oxygen intermediates, thus yielding higher mass activity than their Ir‐based counterparts.^[^ ^14^ ^]^ Moreover, the catalytic reaction pathways of Ru‐based catalysts differ from those of the Ir‐based catalysts, thereby facilitating the design of high‐performance acidic OER catalysts. Ru‐based oxide catalysts predominantly engage in the lattice oxygen‐involved mechanism (LOM) owing to the strong covalent nature of the Ru–O bonds.^[^ ^15^ ^]^ However, the increased activity of Ru‐based oxides via the LOM pathway frequently decreases the stability.^[^ ^16^ ^]^ Typically, amorphization or dissolution occurs on the surface of Ru‐based oxides when oxygen vacancies are not filled by bulk oxygen transfer or by water adsorption with the lattice oxygen involved in the redox reaction.^[^ ^9a^ ^]^ Furthermore, most non‐noble transition metal catalysts that exhibit excellent OER performance in alkaline electrolyzers do not perform satisfactorily in PEMWE because of the leaching of the non‐noble transition metals in acids. Therefore, efficient and stable acidic OER catalysts, which are also inexpensive and nontoxic, must be developed for subsequent industrial applications. Furthermore, considering the usage scenario of PEMWEs, the coupled primary energy usually outputs fluctuating power (wind, photovoltaic energy) because of changing weather, which is a great challenge for the catalysts, especially the anode electrode.^[^ ^17^ ^]^ Kojima et al. reported that overcome several issues, especially input fluctuating power, cell temperature changes, and the degradation of Nafion membranes, related to the direct use of fluctuating power, is the key toward the achievement of a sustainable hydrogen‐based society.^[^ ^17b^ ^]^ This work emphasized that the universal accelerated degradation test (ADT) should be constructed as soon as possible, to clarify the performance degradation mechanism of each component, for rapidly develop efficient and durable catalysts to scale produce green hydrogen. The universal ADT protocol is conducive to simulating the electrolytic cell system in the laboratory and the fluctuating power at the plant level, so as to develop high‐efficiency catalysts according to the actual situation to the maximum extent.

**Figure 1:**
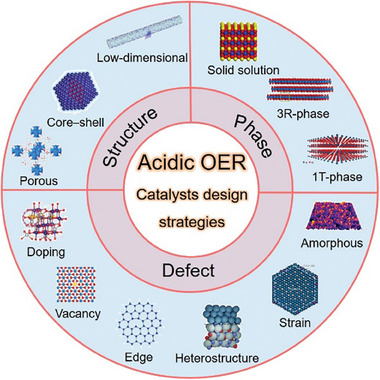
Common strategies to design electrocatalysts for acidic OER.

This review comprehensively outlines strategies involving defect, phase, and structure engineering for the development of acidic OER catalysts with high activity and stability across various scales ranging from atomic to mesoscale nanostructures. The specific roles of sites within or on the electrocatalysts during the acidic OER are delineated. First, the intrinsic link between material defects and catalytic performance is elucidated, underscoring the advantages of defect engineering in catalyst synthesis to satisfy industrial requirements. Subsequently, recent advancements in state‐of‐the‐art acidic OER catalysts and their associated modification strategies are surveyed. The discussion encompasses the creation of defective sites, synthesis of novel active phases, and development of appropriate structures for water splitting, focusing on mass or ion transformation to enhance activity and stability (**Figure** **1**). From an engineering perspective, this review also summarizes mesoscale nanostructures that are conducive to efficient mass transfer, which can potentially promote the industrialization of hydrogen production. The review concludes by highlighting the critical challenges, directing attention to areas of concern, and providing reasonable suggestions with a targeted approach.

## 2 Defect Engineering

Defect engineering has garnered significant attention in materials science, serving as a pivotal methodology for finely adjusting material properties in accordance with the requirements for various applications.^[^ ^4b^ ^]^ In addition to its role in property manipulation, defect engineering is critical for improving material performance across diverse applications, including catalysis, energy storage, and sensing. For example, in catalysis, defective domains exhibit dual functionality, that is, altering and actively participating as catalytic sites; consequently, both catalytic activity and selectivity are substantially increased.^[^ ^1b^ ^]^ The strategic localization of defects within or on materials typically confers properties that markedly differ from those exhibited by the bulk material; this presents avenues for advanced material functionality and purpose‐driven design.

During EWS, the deliberate introduction of defects into the catalysts strongly influences the charge distribution at the active centers, resulting in the i) optimization of intermediate adsorption and desorption behavior, ii) facilitation of rapid electron transfer, iii) adjustment of the d‐band center, and iv) reduction of the energy barrier for the rate‐determining step (RDS); these factors enhance the catalytic performance in water splitting.^[^ ^2^, ^18^ ^]^ In the acidic OER, defective materials must exhibit enhanced activity in addition to stability and long‐term functionality in harsh catalytic environments.^[^ ^19^ ^]^ Defect engineering has the potential to enhance the activity of electrocatalysts for the acidic OER. Commonly used metal oxides, such as iridium oxide (IrO_x_), ruthenium oxide (RuO_x_), cobalt oxide (CoO_x_), and manganese oxide (MnO_x_), serve as OER electrocatalysts in acidic media.^[^ ^20^ ^]^ Deliberately integrating controlled defects, including vacancies or surface terminations, within these materials markedly enhances their OER activity and stability.^[^ ^10^, ^18^, ^21^ ^]^ In addition, hybrid materials combining defect‐engineered metal oxides with substances such as graphene or carbon nanotubes (CNTs) have been explored.^[^ ^22^ ^]^ These hybrid constructs offer improved catalytic activity, stability, charge transfer, and mass transport properties.

In summary, defect engineering has emerged as a promising strategy to enhance the performance of acidic OER electrocatalysts by modifying their electronic and structural attributes. The advancement of defect‐engineered materials for OER electrocatalysis can enable the production of clean and sustainable hydrogen fuels through water splitting, thus approaching a major milestone in the utilization of sustainable energy sources. This section introduces the proposed defect classification system and examines its influence on the performance of the acidic OER.

### 2.1 Point Defects

#### 2.1.1 Doping

Doping defects are point defects originating from the introduction of impurities into the crystalline lattice of a material.^[^ ^4^, ^23^ ^]^ Doping is performed through the incorporation of impurity atoms into the lattice structure during crystal growth or via ion implantation.^[^ ^24^ ^]^ These impurity atoms may replace the host atoms within the lattice, resulting in substitutional doping, or may occupy interstitial positions, resulting in interstitial doping. Consequently, doping mechanisms are intricately linked to vacancy defects. Defect doping is significant in materials science and engineering as it enables the precise modification and regulation of material properties at the atomic level.^[^ ^25^ ^]^

**Figure 2:**
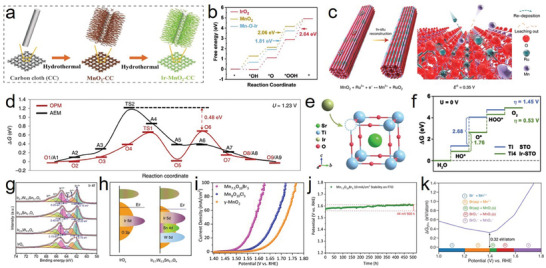
a) The synthesis procedure for the electrocatalysts. b) Gibbs free‐energy diagram for IrO_2_, MnO_2_, and Ir‐MnO_2_. a,b) Reproduced with permission.^[^ ^27^ ^]^ Copyright 2023, Wiley‐VCH. c) Schematic illustration for the in‐situ reconstruction process of Ru/MnO_2_. E^0^ represents the standard redox potentials at 298.15 K and a pressure of 1 atm. d) The free energy (Δ*G*) diagrams of AEM and OPM at 1.23 V versus RHE. States O1–O9 and A1–A9 present the different elementary states in the OPM and AEM pathways, respectively. c,d) Reproduced with permission.^[^ ^28^ ^]^ Copyright 2021, Springer Nature. e) Schematic illustration for the incorporation of Ir dopants into the STO matrix. f) Gibbs free energy diagrams for OER on Ti site in STO (blue), and Ti4 site in Ir‐STO (green). e,f) Reproduced with permission.^[^ ^29^ ^]^ Copyright 2019, Wiley‐VCH. g) Ir 4f spectra for IrO_x_, Ir_0.8_W_0.2_O_x_, Ir _0.9_Sn_0.1_O_x_, and Ir_0.7_W_0.2_Sn_0.1_O_x_. h) Schematic diagram of band structure for IrO_x_ and Ir_0.7_W_0.2_ Sn_0.1_O_x_. g,h) Reproduced with permission.^[^ ^31^ ^]^ Copyright 2022, Wiley‐VCH. i) LSV curves of different catalysts at 1 mV s^−1^ scan rate with iR correction. j) Chronopotentiometry curves (On FTO) of Mn_7.5_O_10_Br_3_ at 10 mA cm^−2^ (25 °C). k) Calculated Pourbaix decomposition free energy (ΔG_pbx_) of Mn_7.5_O_10_Br_3_ from the potential 1.0–1.8V vs RHE at pH 0. The projection of Δ*G*_pbx_ onto the potential axis shows the stable species at the corresponding regions. i–k) Reproduced with permission.^[^ ^36^ ^]^ Copyright 2022, Springer Nature.

Numerous studies on the acidic OER have highlighted the effectiveness of heteroatom doping in optimizing local electronic structures and coordination environments, particularly within materials such as IrO_2_ and RuO_2_.^[^ ^26^ ^]^ Weng et al. reported that high‐valence‐manganese promoted the strong anchoring of Ir species to form Ir atom arrays on α‐MnO_2_ with Ir–O–Mn coordination (**Figure** **2**a), exhibiting excellent activity and stability for acidic OER owing to the strong catalyst–support interaction.^[^ ^27^ ^]^ This study confirmed that the formation of the Ir–O–Mn coordination structure optimized the local electronic structure of the catalyst, thus reducing the adsorption strength between the catalyst and *OOH intermediates (Figure 2b). Similarly, Lin et al. reported a Ru/MnO_2_ catalyst wherein cation exchange occurred, and small Ru ensembles were reconstructed into large patches of Ru atom arrays on the support, which prevented metal leaching and catalyst deactivation (Figure 2c).^[^ ^28^ ^]^ Ru/MnO_2_ showed excellent acidic OER performance with an ultralow overpotential of 161 mV at 10 mA cm^−2^ and remarkable stability for 200 h at the same current density. According to the density functional theory (DFT) calculations, the barrier of AEM was 0.48 eV higher than that of OPM, indicating that the OER pathway at the interface preferentially triggered OPM (Figure 2d). This study confirmed that the in‐situ leaching and recapture of the active sites reconstructed the active center and resulted in a satisfactory catalytic effect. However, cation exchange can still provide a suitable catalytic effect without triggering the conditions for reconstruction. For example, Liang et al. designed Ir‐doped SrTiO_3_ (Ir‐STO) to address cost, efficiency, and stability issues, providing a useful strategy for practical applications.^[^ ^29^ ^]^ They utilized the similar ionic radii of Ir^4+^ and Ti^4+^ doped in perovskite STO to form a solid‐solution material, Ir‐STO (Figure 2e). Ir‐STO exhibited a low noble metal content, acid resistance, and high acidic OER activity. Particularly, the intrinsic inert Ti sites were activated by Ir doping, thus optimizing the Ti^4+^ electronic structure and promoting the conductivity of the materials (Figure 2f). Compared with commercial IrO_2_, the Ir dosage for Ir‐STO was reduced by 57%, whereas the mass activity was increased by 34 times, and it was stable for more than 20 h (at 10/30 mA cm^−2^) under chronoamperometry.

In addition, doping can effectively inhibit the excessive oxidation of noble metals, and the synergy between the heteroatoms increases the durability of the catalyst. For example, Xue et al. demonstrated that S doping significantly weakened the adsorption energy between the *OOH intermediate and Ru sites and efficiently stabilized the lattice oxygen of RuO_2_, thereby enhancing the stability of the catalyst.^[^ ^30^ ^]^ He et al. reported that IrO_2_ co‐doped with W and Sn reduced the valence state of Ir (<4+) through multistage charge redistribution (Figure 2g) and prevented overoxidation at high potentials (>1.6 V vs. RHE); this improved the performance (236 mV @ 10 mA cm^−2^_geo_) and stability (>220 h @ 500 and 1000 mA cm^−2^) of the catalyst for acidic OER.^[^ ^31^ ^]^ Furthermore, W and Sn co‐doping enabled the d‐band center of Ir to approach the Fermi level (Figure 2h), enhancing the binding energies of the oxo‐intermediates with Ir sites and decreasing the energy barrier of the acidic OER, which further accelerated the overall kinetics. Similarly, Sun et al. reported that the 5d‐orbital state of Ir (t_2g_^5^e_g_^0^) in IrO_2_ was modified by Cu doping, resulting in partial oxygen defects owing to the strong Jahn–Teller effect; thus, the IrO_6_ octahedral geometric structure changed, and the degeneracy of the t_2g_ and e_g_ orbitals lifted.^[^ ^26b^ ^]^ This study indicated that doping with transition metals could effectively regulate the orbital state of IrO_2_ by tuning the electron occupation to satisfy the requirements of acidic OER. Chen et al. demonstrated that the d‐band center (E_d_) of RuO_2_ remained away from the Fermi level (E_f_) after Mn doping.^[^ ^23c^ ^]^ Particularly, the electronic structure was optimized by Mn doping, thus causing a weak interaction between the O intermediates and active centers, which is beneficial for acidic OER catalytic kinetics. Similarly, Liu et al. reported that Nd‐doped RuO_2_ considerably weakened the covalence of Ru–O bonds by forming strong Nd–O bonds to extend the durability of the catalyst; moreover, the d‐band center of Ru was moderately reduced to balance the adsorption and desorption of oxo‐intermediates, thus enhancing catalytic activity.^[^ ^16a^ ^]^ Jin et al. reported that Pt doping efficiently prevented the overoxidation of Ru (IV) because of the transfer of electrons from Pt to Ru.^[^ ^26a^ ^]^ Huo et al. demonstrated that doping Re into the IrO_2_ lattice suppressed Ir dissolution, and this was attributed to strong interactions between Re and Ir.^[^ ^32^ ^]^ Lv et al. reported that N/C‐doped IrO_2_ exhibited excellent acidic OER performance owing to the low electronegativity of the N/C atoms, distinctive amorphous structure, and electron enrichment of the active sites.^[^ ^33^ ^]^ Liu et al. confirmed that the presence of Si around Ru sites as an electron reservoir substantially increased the oxidation resistance of the Ru center, thus enhancing its stability in acidic media.^[^ ^34^ ^]^

Unstable non‐noble metal catalysts can be made more suitable for acidic OER operations by doping with non‐metallic atoms.^[^ ^35^ ^]^ Pan et al. developed a low‐cost and stable manganese oxybromide (Mn_7.5_O_10_Br_3_) catalyst, exhibiting excellent OER performance in an acid electrolyzer with a low overpotential of 295 ± 5 mV at 10 mA cm^−2^ and maintaining good stability for >500 h (Figure 2i,j).^[^ ^36^ ^]^ This study demonstrated that the self‐oxidized surface of Mn_7.5_O_10_Br_3_ with enhanced electronic transmission capacity was the primary reason for both high activity and long‐term stability during OER operation (Figure 2k). A densely packed oxide surface with a unique structure formed by this self‐oxidation optimized the binding energies with the OER intermediates. Similarly, Li et al. reported that low‐coordination Ru sites exhibited excellent performance owing to the replacement of O by a partial Cl atom, thus causing the rapid formation of O* from OH*.^[^ ^37^ ^]^ Furthermore, the strong Mn–halogen interactions in Mn_7.5_O_10_Br_3_ markedly affected the Mn‐oxide electron structure and promoted the electron transfer ability of the catalyst, thus enhancing the acidic OER activity. Ou et al. doped the Co_3_O_4_ lattice with a low content of Ir (2.88 wt%), which optimized the electronic structure of the catalyst, created a local stable bonding environment, and enhanced the activity and stability of Co_3_O_4_ during the OER in an acidic electrolyte.^[^ ^24a^ ^]^

Several studies have demonstrated that doping can alter the reaction mechanism at the interface. For example, rutile RuO_2_ tends to catalyze the OER through the LOM mechanism; however, the LOM mechanism rapidly leads to the gradual decline and even disintegration of RuO_2_. According to Wen et al., when atomic W was doped into RuO_2_ without lattice evolution, the formation of Ru–O_bri_–W Brønsted acid sites optimized the energy barrier of the acidic OER and accelerated the overall kinetics via the bridging‐oxygen‐assisted deprotonation mechanism.^[^ ^38^ ^]^ This study demonstrated that the added Brønsted acid sites optimized the proton adsorption energy at the bridging oxygen sites, increased the proton transfer rate on the surface of the catalyst, and resulted in a rapid bridging‐oxygen‐assisted deprotonation, thus accelerating the acidic OER kinetics. Wu et al. demonstrated that doping Ni into the lattice of RuO_2_ considerably stabilized the surface Ru and subsurface oxygen, increasing the stability of the catalyst by an order of magnitude.^[^ ^39^ ^]^ Based on DFT calculations and isotope ^18^O‐labeling, this study confirmed that the AEM occurred on the RuO_2_ (110) surface rather than the LOM. Ni stabilized the O species in the subsurface layer and the Ru species on the surface, which was instrumental in improving the stability of RuO_2_ during the acidic OER. This study surpasses the limits of conventional thermodynamics and proposes new ideas.

**Figure 3:**
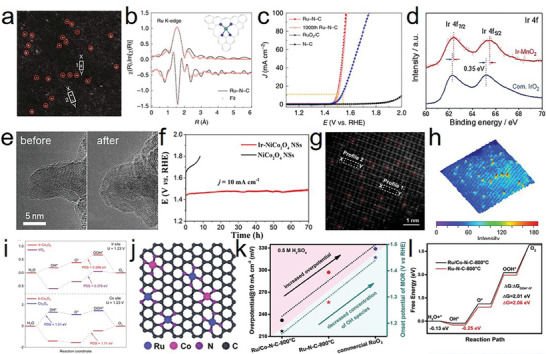
a) Magnified HAADF‐STEM image of Ru‐N‐C. b) The R‐space curve‐fitting of ex situ Ru‐N‐C. Top and bottom curves are magnitude and imaginary part, respectively. Insert shows the structure of the Ru site in Ru‐N‐C. The balls in gray, blue, and light green represent C, N, and Ru atoms, respectively. c) Electrocatalytic OER performances of the Ru‐N‐C and commercial RuO_2_/C in 0.5 m H_2_SO_4_ electrolyte. a–c) Reproduced with permission.^[^ ^40^ ^]^ Copyright 2019, Springer Nature. d) Ir 4f XPS spectrum of Ir‐MnO_2_ and commercial IrO_2_. e) HRTEM images of Ir‐MnO_2_ before and after chronopotentiometry test, no surface amorphization can be seen. d,e) Reproduced with permission.^[^ ^41^ ^]^ Copyright 2021, Elsevier. f) Chronoamperometric response of NiCo_2_O_4_ and Ir‐NiCo_2_O_4_ NSs for OER at 10 mA cm^−2^ in 0.5 m H_2_SO_4_. Reproduced with permission.^[^ ^23a^ ^]^ Copyright 2020, ACS Publications. g) The enlarged area in HAADF‐STEM, with the Ir single atoms marked in circles. h) 3D atom‐overlapping Gaussian function fitting mapping of the selected area. i) The Gibbs free energy diagrams of the four‐electron OER process on the Ir sites and Co sites of these catalysts under the applied overpotentials of 1.23V vs. RHE, respectively. g–i) Reproduced with permission.^[^ ^43^ ^]^ Copyright 2022, Springer Nature. j) Proposed structural model of Ru/Co‐N‐C‐800 °C. k) The relationship between OER performance and the concentration of OH intermediates for Ru/Co‐N‐C‐800 °C, Ru‐N‐C‐800 °C, and commercial RuO_2_. l) OER free energy diagram for Ru/Co‐N‐C‐800 °C and Ru‐N‐C‐800 °C. j–l) Reproduced with permission.^[^ ^47^ ^]^ Copyright 2022, Wiley‐VCH.

Yao et al. dispersed atomic Ru on phosphor‐decorated carbon nitride (Ru–N–C) via simple impregnation and annealing. The morphology and coordination structure are shown in **Figure** **3**a,b.^[^ ^40^ ^]^ This study revealed that isolated Ru atoms fixed by the surrounding N atoms stabilized the catalyst through the contraction of Ru–N bonds when the catalyst was employed under oxidation conditions in an acidic corrosion environment. During the operation, the average bond length of Ru–N/O bond (2.05 Å) was shorter than that of Ru–N (2.08 Å) in the ex‐situ sample, and the fitted Ru–N bond distance was larger than that of the Ru–O bond, thus demonstrating the strong interaction and hybridization of Ru–O coordination. The shrinkage of the Ru–N bonds provided additional energy to immobilize the Ru atom, thus preventing possible dissolution and overoxidation. The Ru–N–C catalyst exhibited remarkable intrinsic activity with a low overpotential of 267 mV at 10 mA cm^−2^, mass activity of 3571 A g_metal_^−1^, and turnover frequency (TOF) of 3348 O_2_ h^−1^ (Figure 3c). Moreover, Ru–N–C remained stable after 30 h of operation without considerable deactivation or decomposition. This study also demonstrated that the formed O–Ru_1_–N_4_ sites under operando conditions were responsible for the high OER performance and long‐term stability; the dynamic adsorption of a single oxygen atom on the Ru site was verified under OER operating conditions based on operando synchrotron radiation X‐ray absorption spectroscopy and infrared spectroscopy. Shi et al. investigated single Ir‐site doping on γ‐MnO_2_ (Ir‐MnO_2_) and observed a low overpotential of 218 mV at 10 mA cm^−2^ and excellent TOF (7.7 s^−1^).^[^ ^41^ ^]^ Considering the Ir 4f spectra, Ir‐MnO_2_ exhibited a positive shift (0.35 eV) compared with commercial IrO_2_ (Figure 3d), indicating an increased valence of Ir. The electron cloud density around the Ir sites decreased, resulting in a strong Ir–O interaction, which was confirmed by X‐ray absorption near edge structure spectra (XANES). The high‐resolution transmission electron microscopy (HRTEM) images showed no distinct structural reconstruction after the durability test, because the lattice oxygen oxidation was activated (Figure 3e). Xi et al. reported similar results.^[^ ^23a^ ^]^ Particularly, atomic Ir and the incorporation of NiCo_2_O_4_ with abundant oxygen vacancies enhanced the OER performance in acidic media, with remarkable durability (Figure 3f). Ir doping enhanced the activity of low‐coordinated Co sites near the oxygen vacancies, thereby facilitating surface electronic exchange and transfer, which optimized the OER performance.

The coordination environment of active atoms plays an important role in catalysis. Some studies have indicated that the coordination number of the active center atoms is closely related to the construction of the space structure of the corresponding active sites, thus affecting the catalytic stability.^[^ ^42^ ^]^ In many studies on atomic dispersion catalysts (ADCs), the interactions between the active atoms and the matrix have been found to affect the activity and stability of the catalysts. For example, Zhu et al. employed a mechanochemical approach to prepare Ir‐doped Co_3_O_4_, which exhibited excellent performance, with 236 mV at 10 mA cm^−2^ and chronopotentiometric stability of 30 h.^[^ ^43^ ^]^ The developed Ir–O–Co structure was regarded as the active site for the acidic OER. Furthermore, the isolated Ir atom effectively increased the conductivity of the catalyst and optimized the energy barrier between the catalyst and the oxygen intermediates (Figure 3h,i). Other studies showed that the electronic structure of Ru modified by the strain of the Pt_skin_ shell,^[^ ^44^ ^]^ atomic Co bound with heteropyridinic‐/amino‐N ligands (HNC‐Co),^[^ ^45^ ^]^ and single Pt immobilized by carbon nitride materials (Pt_1_‐C_2_N_2_)^[^ ^46^ ^]^ also demonstrated superior OER performance. Moreover, atomic Co sites (Co‐N4) efficiently redistributed the electronic structure of atomic Ru and optimized the bonding strength of the intermediate species with the Ru sites (Ru‐N4), thus enhancing the activity (Figure 3j–l).^[^ ^47^ ^]^

The active atoms within ADCs generally demonstrate excellent performance because their electronic structures are modified upon bonding with the surrounding atoms. Consequently, this interaction forms a robust coordination structure within the matrix. Furthermore, the modified sites substantially change the binding energy between the active centers and the intermediates.^[^ ^48^ ^]^ However, further investigation is required to accurately understand the active mechanism of these catalytic reactions. Careful analysis is necessary to determine whether the choice of carrier is consistent with the requirements of the catalytic reactions. Some acid‐stable materials such as MnO_2_ and TiO_2_ have been used as carriers to anchor Ir or Ru atoms. The optimized electronic structure of the active center provides satisfactory activity and stability for the acidic OER.^[^ ^27^ ^]^ However, the potential tolerance of MnO_2_ is limited, and it cannot fulfill the conditions for its stability under a high oxidation potential; therefore, it is typically not favorable for the development of high‐current catalysts.^[^ ^20^, ^41^ ^]^ Furthermore, some crystalline metallic materials, including carbon‐based materials, have limitations such as dissolution and deterioration, which cause catalyst disassembly.^[^ ^49^ ^]^

However, owing to the characteristics of different atoms, doping generally generates various defects in composite materials, such as vacancies^[^ ^23^, ^50^ ^]^ and lattice strain,^[^ ^15^, ^51^ ^]^ which can further promote catalytic activity, as described in sections 2.1.2 and 2.3.1.

#### 2.1.2 Vacancies

Vacancy defects are point defects that appear when a lattice site remains unoccupied by an atom or ion. In electrocatalysis, these vacancies can determine the catalytic activity of the materials.^[^ ^2^, ^52^ ^]^ Vacancy defects influence the electronic structure of a material and generate new electronic states that actively participate in catalytic reactions.^[^ ^53^ ^]^ Furthermore, vacancies are instrumental in altering the surface reactivity of the material by creating new active sites and modifying the adsorption and desorption behavior of reactants and intermediates.^[^ ^54^ ^]^ Additionally, vacancies markedly affect atomic diffusion and mobility within the lattice, thereby directly influencing the kinetics of the catalytic reactions. Hence, vacancy defects must be effectively investigated and controlled to facilitate the design and optimization of electrocatalysts for diverse applications, including energy conversion, storage, environmental remediation, and chemical synthesis. Notably, with reference to the acidic OER, vacancies induce substantial alterations in the electronic structure of the active centers or atoms; however, excessive vacancies tend to limit the activity and stability of the catalysts.^[^ ^55^ ^]^ Excessive vacancies within a material can render its overall structure thermodynamically unstable, potentially resulting in phase transformations or disintegration. In contrast, a low concentration of vacancies may not distinctly enhance the performance. Therefore, the synthesis of active materials with precisely controlled numbers and types of vacancies remains an ongoing research area.

**Figure 4:**
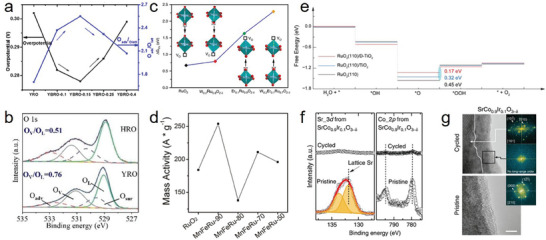
a) The relationships between overpotential (at 10 mA cm^−2^) and O‐Vcancies concentration. Reproduced with permission.^[^ ^55^ ^]^ Copyright 2020, ACS Publications. b) XPS characterization (O 1s) for HRO and YRO. Reproduced with permission.^[^ ^57^ ^]^ Copyright 2022, Royal Society of Chemistry. c) Calculated free energies for oxygen vacancy formation (V_O_) of different RuO_2_‐based electrocatalysts, illustrating that the co‐doping of W and Er into the RuO_2_ lattice is beneficial to suppress oxygen vacancy formation. V_O_ for W_0.2_Er_0.1_Ru_0.7_O_2‐δ_ exceeds the free energy of the redox couple H_2_O/O_2_ (1.23 eV) by far, implying that this catalyst is not prone to degradation under the harsh anodic operation conditions in the acidic oxygen evolution reaction (OER). Reproduced with permission.^[^ ^58b^ ^]^ Copyright 2021, Wiley‐VCH. (d) Lines showing the mass activity of RuO_2_ and all co‐doped composites at 1.7 V vs RHE. Reproduced with permission.^[^ ^58a^ ^]^ Copyright 2020, ACS Publications. e) Calculated free‐energy diagrams at 1.5 V for RuO_2_ (110) (black line) and that supported on either TiO_2_ (blue line) or D‐TiO_2_ substrate (red line). Reproduced with permission.^[^ ^54^ ^]^ Copyright 2022, ACS Publications. f) Sr 3d and Co 2p XPS for SrCo_0.9_Ir_0.1_O_3−δ_ before and after the electrochemical tests. g) HRTEM images of pristine and cycled SrCo_0.9_Ir_0.1_O_3−δ_ (by 5 cycles, scale bar, 5 nm). f,g) Reproduced with permission.^[^ ^60^ ^]^ Copyright 2019, Springer Nature.

O vacancies generally optimize the energy barrier of O‐containing intermediates binding with catalysts, thereby enhancing the activity and reducing the binding strength with oxo‐intermediates during the acidic OER.^[^ ^56^ ^]^ Vacancies are commonly generated by doping and/or replacing heteroatoms in active centers. For example, Wang et al. synthesized Y_2−x_Ba_x_Ru_2_O_7_ with abundant O vacancies by partially replacing Y^3+^ with Ba^2+^, which improved the OER performance in 0.5 m H_2_SO_4_.^[^ ^55^ ^]^ This study confirmed that doping with the low‐valence metal Ba caused the appearance of O vacancies and further affected the valence state of Ru, resulting in stronger electrophilicity of the catalysts and an improved deprotonation rate during the OER. Moreover, the amount of elemental doping was reported to be closely related to the number of oxygen vacancies, which affected the catalytic performance (**Figure** **4**a). Although the amount and species of doping elements and the performance improvement are not directly correlated, further clarification is required. Yan et al. demonstrated that the formation energy and number of O vacancies were controllable by highly electronegative Ho, which replaced A‐site atoms in Y_2_Ru_2_O_7−δ_, thus increasing the strength of Ru–O bonds and preventing the dissolution of Ru (Figure 4b).^[^ ^57^ ^]^ This study provided a new strategy for forming and modifying the local structure of the O vacancies. Hao et al. (Wu et al.) reported similar results, where outstanding performance was achieved by co‐doping W and Er (Mn and Fe) into RuO_2_, resulting in a higher activity than that of the commercial catalyst (Figure 4c,d).^[^ ^58^ ^]^ Apart from generating active oxygen vacancies and optimizing the electronic structure, studies on element‐doped catalysts should also consider whether the crystal structure can be used as a consistent reference before and after doping, which is generally easily ignored. Gong et al. demonstrated that Co‐doped RuO_2_ formed abundant oxygen vacancies and caused lattice contraction, thus optimizing the antibonding states of the adsorbed O species and Ru sites, which reduced the free energy in the RDS and stabilized the lattice oxygen during the OER.^[^ ^59^ ^]^ However, considering multiple variables make it difficult to confirm the actual active site and its working mechanism, which must be distinguished. Wang et al. demonstrated that TiO_2_ enriched with oxygen vacancies dispersed active RuO_2_ and regulated the electronic structure of the active centers.^[^ ^54^ ^]^ The continuous band structure at the interface between the defective support TiO_2_ and active RuO_2_, and the low energetic barrier for *OOH formation, were responsible for improving the acidic OER kinetics (Figure 4e). Furthermore, the materials underwent easier phase transformation, surface reconstruction, and even disintegration when the O vacancies were triggered. The surface of SrCo_0.9_Ir_0.1_O_3−δ_ was reconstructed with Sr and Co leaching during electrochemical cycling (Figure 4f,g).^[^ ^60^ ^]^ The formation of corner‐shared and undercoordinated IrO_x_ octahedra was responsible for the higher activity observed. Similarly, Jaramillo et al. demonstrated Sr leaching from the surface of SrIrO_3_ and the formation of a highly active surface for the acidic OER.^[^ ^61^ ^]^

The oxygen‐vacancy‐rich reaction interface is a crucial factor in OER catalysis, considering that the center of the O 2p band is close to the Fermi level, which can accelerate charge transfer and reduce the reaction energy barrier. However, the stability of the interface, even for catalysts with abundant oxygen vacancies under rapid reaction cycles, must also be considered. Wang et al. reported that Rh doping could efficiently provide stable O vacancies for sustainable acidic OER catalysis.^[^ ^62^ ^]^ In addition, Yan et al. demonstrated that the strong electronic coupling between RuO_2_ and NC/CNTs in the presence of O vacancies enhanced the catalytic activity of Ru and the stability of lattice O and surface Ru.^[^ ^22a^ ^]^

Apart from anionic O vacancies, which preferentially trigger LOM to accelerate kinetics, metal cation vacancies markedly affect the acidic OER. Cation vacancies, particularly in low‐dimensional materials, facilitate rapid proton diffusion and mass transfer, which can further improve the current density in PEM. For example, Fan et al. synthesized a new 3R phase IrO_2_ with abundant Ir vacancies, achieving an ultralow overpotential of 188 mV at 10 mA cm_geo_^−2^ and a high TOF of 5.7 s_UPD_^−1^ at 1.5 V vs. RHE.^[^ ^63^ ^]^ This study indicated that the new active sites at the edge structure in 3R‐IrO_2_ and rapid proton transportation through Ir vacancies were responsible for the enhanced OER performance under acidic conditions. Natarajan et al. illustrated a similar situation for Co_3_O_4_.^[^ ^64^ ^]^ This study confirmed that the number of Co^3+^ vacancies substantially increased the Co^2+^/Co^3+^ ratio, and that the exposed Co^2+^‐rich surface enhanced the activity and stability for the acidic OER, which is consistent with the findings reported by Yan et al.^[^ ^65^ ^]^ Moreover, alkaline etching is a general method used for the synthesis of metal vacancies.^[^ ^66^ ^]^ Wang et al. demonstrated that metal cation vacancies and the relocated atoms effectively modified the d‐band center and electronic structure, thus improving the acidic OER performance and kinetics.^[^ ^66b^ ^]^

The formation of vacancies considerably influences the electronic structure and charge redistribution of the active centers. However, the abundance of vacancies is closely related to lattice parameters; consequently, the durability of the catalyst is affected.^[^ ^52^ ^]^ Therefore, establishing a dynamic balance between abundant vacancies and catalytic activity/stability remains a crucial research objective.

### 2.2 Plane Defects

#### 2.2.1 Edge

**Figure 5:**
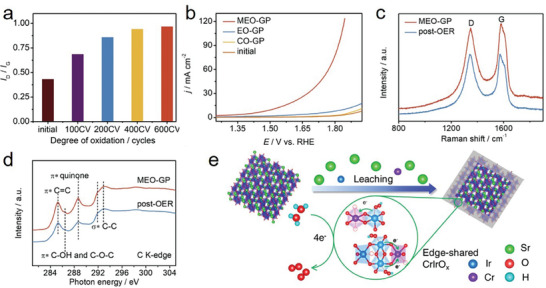
a) The *I*_D_/*I*_G_ ratios from the Raman spectra of GP during 600 cycles of CV oxidation. b) LSV curves of initial GP and GP activated by different methods. c) Raman spectra of MEO‐GP before and after OER test. d) Soft C K‐edge spectra of MEO‐GP before and after OER test. a–d) Reproduced with permission.^[^ ^68^ ^]^ Copyright 2022, Elsevier. e) Surface reconstruction of BCC‐Cr‐SrIrO_3_. Reproduced with permission.^[^ ^74^ ^]^ Copyright 2022, Elsevier.

Generally, the edge regions of catalysts exhibit distinctive and typically unexpected performance owing to their uniform electronic structure and rapid molecular/ion diffusion.^[^ ^67^ ^]^ The synthesis of catalysts with substantially exposed active edge sites is a promising strategy for enhancing the catalytic efficiency. Furthermore, refining the catalytic activity at the edges involves the deliberate modulation of the electronic structure to align precisely with the target reaction requirements. For example, Lu et al. revealed that oxygen‐containing functional groups inhibited the oxidation of graphite carbon and accelerated the kinetics of the acidic OER.^[^ ^68^ ^]^ In this study, a phenanthrenequinone‐like (PQ‐like) moiety formed at the edge of a graphite flake (GP) after mild electrochemical oxidation (MEO) treatment was theoretically and experimentally confirmed to be the most active and stable species; its performance was superior to that of the commercial benchmark IrO_2_/RuO_2_, with a low overpotential of 270 mV at 10 mA cm^−2^. The degree of defects in carbon can be detected by Raman spectroscopy (**Figure** **5**a); the I_D_/I_G_ ratio increased with the number of cycles, indicating that the number of defects in the material increased. The study demonstrated the advantages of MEO over other oxidation methods (Figure 5b), with excellent acidic OER performance. This was primarily because carbon materials prepared by other oxidation methods did not contain highly active PQ‐like groups. The PQ‐like moiety of MEO‐GP was structurally stable after 320 h of continuous operation, with a current density of 20 mA cm^−2^. The I_D_/I_G_ ratio minimally changed (Figure 5c), and the active species remained as PQ‐like moieties (Figure 5d) after the long‐term stability test. Therefore, the PQ‐like moiety at the edge of MEO‐GP can remain stable during long‐term acidic OER. This study provides a new approach to analyze the active species of carbon materials in electrochemical catalytic processes at the molecular scale; the functional group conversion mechanism has also become a new theoretical basis for other electrochemical oxidation reactions.

Carbon edge defects have been modified through different methods, such as fixing active atoms, doping, and substitution, for various reactions. For example, Sun et al. developed ultrathin nitrogen‐doped holey carbon@graphene, where a highly active pyridinic‐N doped at the edge of the holey carbon@graphene sheet provided mechanical support and promoted charge transfer, thus improving the acidic OER performance with high mass and charge transfer.^[^ ^69^ ^]^ The operating potential window of the OER has never exceeded the limit because of the nature of carbon materials; however, it remains an important milestone for low‐cost and practical anode catalysts.^[^ ^70^ ^]^ Preventing physical and chemical changes of carbon‐based materials in acidic electrolytes at high oxidation potentials is an important topic for further investigation.

In addition to carbon‐based materials, various active 2D porous nanosheets (NSs) such as RuO_2_ NSs,^[^ ^16^, ^71^ ^]^ Ir‐containing NSs,^[^ ^72^ ^]^ and RuCu NSs^[^ ^73^ ^]^ have been synthesized for the acidic OER; in these materials, additional edge sites are exposed to enhance the activity. The catalytic activity of the edge sites is increased owing to their distinctive electronic structure; however, the dynamic evolution of active edge sites must also be considered. Zhang et al. reported that an amorphous layer shell with abundant edge‐shared IrO_x_ and CrO_x_ octahedrons around body‐centered cubic (BCC)‐Cr–SrIrO_3_, with rapid leaching of Sr, was formed in situ during the acidic OER (Figure 5e).^[^ ^74^ ^]^ In the bimetallic octahedrons, the OER activity of each Ir‐site was increased dozens of times, and the mass activity was 417.6 A g_Ir_^−1^ at an overpotential of 0.3 V, attributed to the synergistic electron coupling effect between Cr and Ir. Particularly, the strong coordination persistence of the Ir sites in the edge‐shared octahedra was found to be closely related to the enhanced durability during operation.

#### 2.2.2 Heterostructure

The interface between electrodes and electrolytes plays a pivotal role in electrochemical reactions.^[^ ^75^ ^]^ Owing to a larger exposed specific surface area, the performance of porous catalysts is superior to that of bulk configurations. This feature improves the catalyst–electrolyte interface, facilitating the proliferation of active sites that drive the reaction.^[^ ^76^ ^]^ However, achieving highly active and stable interfaces suitable for the acidic OER is considerably challenging. Ongoing research efforts are focused on addressing these challenges to develop durable and highly active catalysts.^[^ ^77^ ^]^ The heterostructure optimizes the energy barriers between the catalyst interfaces and reactants/O intermediates, thus promoting the OER.^[^ ^78^ ^]^ Notably, heterostructures alter the original d‐band configuration of the active centers, thereby augmenting catalytic activity and stability.^[^ ^79^ ^]^ These abundant heterostructures within the catalysts expose more edges with increased catalytic activity, providing additional active sites that promote the OER.^[^ ^80^ ^]^ Furthermore, the electron distribution is changed in the heterostructure, consequently tuning the electronic structure of the active centers, reducing the barriers, and enhancing the catalytic performance.^[^ ^81^ ^]^ For the acidic OER, the acid resistance and electrochemical corrosion resistance of the interface must be considered to facilitate interface modifications.

**Figure 6:**
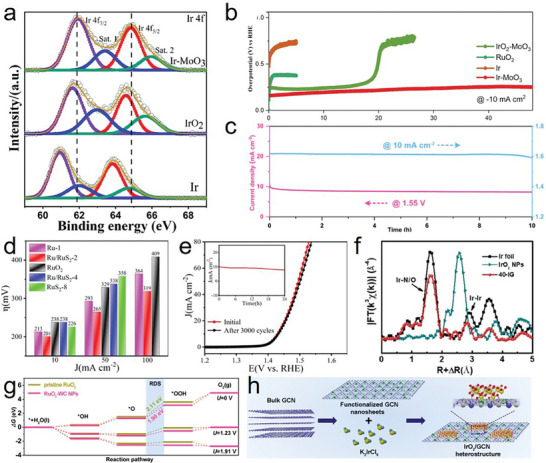
a) HR‐XPS of Ir 4f. b) Chrono‐potentiometric curves of IrO_2_‐MoO_3_, Ir‐MoO_3_, RuO_2_, and Ir. a,b) Reproduced with permission.^[^ ^82^ ^]^ Copyright 2021, Springer Nature. c) Long‐term chrono‐potentiometric and chronoamperometric curves of RuO_2_‐WC NPs at 10 mA cm^−2^ and 1.55 V, respectively. d) corresponding overpotentials of Ru‐1, Ru/RuS_2_‐2, Ru/RuS_2_‐4, RuS_2_‐8, and commercial RuO_2_ catalysts. e) LSV curves before and after 3000 cycles and inserted *i‐t* chronoamperometric response of Ru/RuS_2_‐2. d,e) Reproduced with permission.^[^ ^8^ ^]^ Copyright 2021, Wiley‐VCH. f) Fourier transforms of k^3^‐normalized Ir L_III_‐edge EXAFS of 40‐IG, IrO_2_ NPs, and Ir foil. g) Free‐energy landscape of pristine RuO_2_ and RuO_2_‐WC NPs at zero potential (U = 0), equilibrium potential (U = 1.23 V), and the potential (U = 1.91 V) for which each step is downhill of RuO_2_‐WC NPs, respectively. c,g) Reproduced with permission.^[^ ^85^ ^]^ Copyright 2022, Wiley‐VCH. h) Illustration of the synthesis of IrO_2_/GCN. f,h) Reproduced with permission.^[^ ^87^ ^]^ Copyright 2019, Wiley‐VCH.

Different energy band arrangements of different phases result in charge transfer at the interface, which is beneficial for the surface electronic modulation of the heterostructure. In the acidic OER, a matched heterostructure can reduce the energy barriers of the RDS and optimize the desorption of oxygen intermediates, thus improving the activity and stability of the catalysts. Liu et al. demonstrated that highly electron‐deficient metal–metal oxide heterostructures and the high oxidation state of Ir^x+^ (x > 4) on MoO_3_ markedly enhanced the performance, acid resistance, and durability during acidic OER operation.^[^ ^82^ ^]^ Notably, Ir‐MoO_3_ embedded with graphitic carbon layers with an electron‐deficient surface demonstrated excellent performance with an ultralow overpotential of ≈156 mV at 10 mA cm^−2^ and high durability and current density of 100 mA cm^−2^ for 48 h (**Figure** **6**b). Electrons were transferred from Ir to the strongly electron‐absorbing MoO_3_, and the high‐valence state of Ir accounted for the excellent catalytic activity (Figure 6a). Fan et al. conducted a similar study, in which RuO_2_/Co_3_O_4_–RuCo@NC composites with rich metal–semiconductor interfaces displayed remarkable activity for the acidic OER because of the facilitation of charge transfer and the presence of a carbon coating.^[^ ^83^ ^]^ Shaikh et al. developed a heterostructured material, Ni_3_S_2_@NiSe/NF, via co‐sulfurization and selenization with rapid thermal diffusion.^[^ ^84^ ^]^ The heterostructure facilitated charge transfer and electron localization, thus improving the catalytic performance. Zhu et al. developed a Ru/RuS_2_ heterostructure in a eutectic salt system, which exhibited outstanding performance with a low overpotential of 201 mV at 10 mA cm^−2^ and was stable for 24 h at the same current density (Figure 6d,e).^[^ ^8^ ^]^ The interfacial charge rearrangement and the enhanced conductivity on the Ru/RuS_2_ heterostructure considerably optimized the adsorption of intermediates with surface electron‐deficient Ru atoms at the heterostructure, thus reducing the energy barriers. Such interfacial effects accelerate the kinetics, improve the intrinsic activity, and provide excellent long‐term stability. Similarly, Liu et al. developed IrO_2_/Ir to enhance its intrinsic ability for the acidic OER.^[^ ^66b^ ^]^ Li et al. reported that the Ru d‐band center of a Ru@V–RuO_2_/C HMS catalyst shifted negatively, thus optimizing the binding energy between the catalyst surface and the adsorbed oxo‐intermediates and reducing the overpotential.^[^ ^20e^ ^]^ Moreover, the LSV curve of Ru@V–RuO_2_/C HMS shifted slightly to the positive direction after an accelerated decay test of 5 k cycles, demonstrating that heterostructure can obviously promote corrosion‐resistance and durability.

In addition to directly regulating and optimizing the reaction interface, the interface can assist the reaction without participating in it, for example, by stabilizing the active center through strong interactions between the active center and the carrier. Sun et al. demonstrated that the electronic structure of Ru sites was optimized by strong catalyst–support interactions between RuO_2_ nanoparticles (NPs) and WC (RuO_2_–WC NPs) that reduced the reaction barrier (Figure 6g).^[^ ^85^ ^]^ The most stable RuO_2_ (100) and WC (001) along the [001] direction formed RuO_2_ (100)–WC (001) with high symmetry and the smallest lattice mismatch. The formation energy of RuO_2_–WC heterostructure was calculated to be −0.23 eV atom^−1^, indicating its thermodynamic stability. Moreover, long‐term chronopotentiometry and chronoamperometry (Figure 6c), revealed that the material was stable for 10 h. Huang et al. reported that the introduction of nanocrystalline CeO_2_ into Co_3_O_4_ to form a tight heterogeneous interface substantially enhanced the intrinsic activity of Co_3_O_4_ and inhibited its redox properties during the acidic OER.^[^ ^86^ ^]^ Furthermore, Chen et al. developed an IrO_2_/graphitic carbon nitride (GCN) heterostructure to enhance the acidic OER (Figure 6h).^[^ ^87^ ^]^ Low‐coordinate IrO_2_ NPs and superhydrophilic, highly stable GCN NSs were found to be pivotal for the excellent performance and stability in the acidic OER. Particularly, as IrO_2_ was highly dispersed on GCN, the number of active sites increased, and the superhydrophilic matrix was favorable for the acidic OER. The coordination number of Ir atoms was lower than that of general IrO_2_ because of the strong interaction between IrO_2_ and GCN, thus increasing the electron density around Ir and the lattice strain (Figure 6f); this optimized the binding energies between the oxo‐intermediates and the catalyst and accelerated the kinetics. Shi et al. confirmed that the dynamic migration of oxygen species between IrO_x_ and Nb_2_O_5‐x_ improved the performance by altering the Ir electronic structure and inhibited the excessive oxidation of Ir by transferring excessive O from IrO_x_ to Nb_2_O_5‐x_.^[^ ^88^ ^]^ Hou et al. developed multi‐heterostructure IrO_2_@Ir/Co_3_O_4_ materials, which exhibited excellent activity and long‐term stability owing to the compressed Ir–O and Co–O bonds resulting from electron transfer.^[^ ^89^ ^]^

However, some studies focused only on the catalytic activity of the heterogeneous interface and considered that the electronic structure of the active center was optimized upon being combined with a carrier or another substance. Although this assumption is correct, it is extremely limited, without a comprehensive understanding of the areas in which catalysis is improved at the interface. Moreover, the optimal construction of heterostructures must consider the adaptability of the two original phase materials to the reaction.

### 2.3 Other Effects Derived by Defects

#### 2.3.1 Defects Induced Strain

Lattice strain, which generally originates from lattice vacancies, distortions, or mismatches, is one of the most effective strategies for adjusting the electronic structure of active centers.^[^ ^90^ ^]^ The strain primarily alters interatomic distances, thereby enhancing the performance characteristics.^[^ ^20d^ ^]^ Notably, the direction and magnitude of the lattice strain considerably affect the behavior of the intermediates at the active site. Consequently, the application of this strain has been found to be instrumental in enhancing both the activity and stability of catalysts designed for the acidic OER.^[^ ^91^ ^]^ Qin et al. studied the lattice strain caused by electrochemical Li insertion to improve the OER performance of RuO_2_.^[^ ^92^ ^]^ The electronic structure of Ru was modified, and the intrinsic lattice strain of RuO_2_ was tuned when Li was inserted into the RuO_2_ lattice. Li doping provided electrons for Ru atoms, further reducing the valence state of Ru and indicating that the interaction of Li–O was strong; subsequently, the Ru–O covalency was decreased, confirming that Ru–O 4d–2p hybridization was weakened. Therefore, during the acidic OER, the participation of lattice oxygen was inhibited, which increased the oxidation and dissolution resistance of Ru and further improved the stability of the catalyst. According to Liu et al., the compressive strain of the catalyst was attributed to the smaller atomic radius of Cr that replaced Ir in Cr–IrO_2_/Ir, wherein the relocated atoms caused a d‐band center shift. Subsequently, the binding energy with the intermediate was optimized, and the kinetics were accelerated.^[^ ^66b^ ^]^ Yao et al. reported that the electronic structure of Ru was optimized by the compressive strain from the Pt_skin_ shell, and the d‐band center approached the Fermi level to improve its binding with oxygen, thereby acquiring oxidation and dissolution resistance.^[^ ^44^ ^]^ The Ru_1_–Pt_3_Cu catalyst showed excellent OER performance in acid, with an overpotential of only 220 mV at 10 mA cm^−2^_,_ and its stability was an order of magnitude higher than that of commercial RuO_2_. The existence of multiple grain boundaries in the microstructure generally causes torsional strain in materials and substantially changes their properties. Hao et al. demonstrated that a Ta_0.1_Tm_0.1_Ir_0.8_O_2−δ_ nanocatalyst with numerous grain boundaries exhibited an ultralow overpotential of 198 mV at 10 mA cm^−2^ toward the OER in 0.5 M H_2_SO_4_.^[^ ^51^ ^]^ The synergistic effects between the grain boundaries caused Ir–O torsional strain and doping‐induced ligands; these factors altered the adsorption energy of the oxygen intermediates, as confirmed by X‐ray absorption spectra (XAS) and DFT calculations. Ta_0.1_Tm_0.1_Ir_0.8_O_2−δ_ operated stably at 1.5 A cm^−2^ for 500 h with a low mass loading of 0.2 mg cm^−2^ in the PEM device. Huang et al. observed that s‐RuO_2_/ATO with a high tensile strain efficiently inhibited the overoxidation of Ru.^[^ ^20d^ ^]^ Therefore, the catalyst was operated at 10 mA cm^−2^ over 150 h in three‐electrode system and 0.5 A cm^−2^ for 40 h in a PEM device. Furthermore, the kinetics at the reaction interface could be optimized by precisely adjusting the compression strain effect. Meng et al. demonstrated that the Ir–O bond length could be adjusted by controlling the growth of the IrO_x_ atomic layer through the gradient compressive strain effect of IrCo, and the binding energy between the oxo‐intermediates and the catalyst could be optimized, thus accelerating the RDS to form HOO* from O*.^[^ ^20c^ ^]^ This study provided an approach to accurately regulate the catalyst strain, which could further clarify the acidic OER kinetics and mechanism. However, accurately characterizing and adjusting the degree of strain remains challenging.

#### 2.3.2 Defects Induced Amorphous Structure

Until recently, the prevailing consensus in research was that crystalline structures were superior, owing to their ordered crystallinity and exposed specific facets, displaying excellent performance in various applications. However, studies have indicated that the performance of certain catalysts consisting of amorphous structures with active centers or shells surpasses that of their crystalline counterparts.^[^ ^15^, ^72^, ^93^ ^]^ In contrast to regular surfaces exhibiting long‐range ordered crystal structures, amorphous materials possess atoms in a state of disorder, generally featuring numerous defects such as vacancies and unique active edges. Consequently, these amorphous materials exhibit unexpected properties.^[^ ^94^ ^]^ Wu et al. prepared amorphous Ir NSs by directly annealing iridium acetylacetonate (Ir(acac)_3_) with alkali nitrate (KNO_3_) in air.^[^ ^72^ ^]^ The valence state of Ir in the amorphous Ir NSs increased to less than +4 during the acidic OER and returned to the original state after being tested, as revealed by in‐situ X‐ray absorption fine structure spectra. In addition to monometallic amorphous NSs, various noble‐containing bimetallic and trimetallic NSs, such as RuIr, RhNi, RhCo, RhRu, IrFe, IrNi, IrCo, and IrRhRu NSs, have been developed to enhance the OER performance in acidic media. An et al. reported that amorphous noble metal layers are considerably stable for the acidic OER.^[^ ^93^ ^]^ In addition, Wang et al. investigated the difference between the OER performance of amorphous and crystalline structures with RuTe_2_ porous nanorods.^[^ ^15b^ ^]^ The local distortion–strain effect in the amorphous system was found to promote electron exchange and align with the one‐dimensional porous structure, which facilitated mass transfer and enhanced the OER performance in acidic electrolytes. Liu et al. reported that Li‐ion doping of crystalline IrO_2_ disrupted its uniform and ordered structure to form an amorphous IrO_2_ structure.^[^ ^95^ ^]^ The excellent performance was attributed to the shrunken Ir–O bond and the more electrophilic high‐Ir oxidation state, which increased the OER activity. The flexible valence changes of Ir atoms in the amorphous structure resulted in rapid OER kinetics, as demonstrated by in‐situ XAS and DFT calculations. However, the effect of subsequent Li leaching on IrO_2_ during the OER was ignored; this aspect requires further investigation. Zhang et al. developed a new type of Cr–SrIrO_3_ (BCC‐Cr–SrIrO_3_) for the acidic OER.^[^ ^74^ ^]^ An amorphous layer of CrIrO_x_, with edge‐shared CrO_x_ and IrO_x_ octahedrons, was formed in situ during the OER because of the rapid leaching of Sr over BCC‐Cr–SrIrO_3_. This amorphous layer showed excellent Ir stability in harsh environments because of its strong coordination persistence. Yang et al. reported that the leaching of unstable metals and proton adsorption did not destroy the initial crystal structure.^[^ ^94^ ^]^ Even after Sr^2+^/H^+^ ion exchange in acid and in‐situ structural rearrangement during electrocatalysis, ultrasmall, surface‐hydroxylated, and rutile crystal‐active phases were obtained rather than amorphous IrO_x_H_y_. Notably, the amorphous catalysts or surfaces of the catalysts in these studies can stably exist in the acidic OER because they are highly stable noble metal species, and their strong interaction with O is an important feature that is not possessed by other metals (**Table** **1**).

**Table 1:** Typical catalysts of Defect Engineering for acidic OER.

Defect Engineering	Catalysts	Overpotential [mV] at 10 mA cm^−2^	Stability	Reference
Doping	12Ru/MnO_2_	161	200 h@ 10 mA cm^−2^	Lin et al.^[^ ^28^ ^]^
	Ir_0.7_W_0.2_Sn_0.1_O_x_	236	220 h@ 1 A cm^−2^	He er al.^[^ ^31^ ^]^
	Mn‐RuO_2_	158	10 h@ 10 mA cm^−2^	Chen et al.^[^ ^23c^ ^]^
	Ni‐RuO_2_	214	>200 h@ 10 mA cm^−2^	Wu et al.^[^ ^39^ ^]^
	Ir‐MnO_2_	218	650 h@ 10 mA cm^−2^	Shi et al.^[^ ^41^ ^]^
	Ir‐Co_3_O_4_	236	30 h@ 10 mA cm^−2^	Zhu et al.^[^ ^43^ ^]^
	Ir‐Co_3_O_4_‐NS‐350	226	500 h@ 10 mA cm^−2^	Liu et al.^[^ ^96^ ^]^
	Gd‐IrO_2‐δ_	260	200 h@ 10 mA cm^−2^	Wu et al.^[^ ^97^ ^]^
	Si‐RuO_2_–0.1	226	800 h@ 10 mA cm^−2^	Ping et al.^[^ ^98^ ^]^
	In‐RuO_2_/G	187	100 h@ 100 mA cm^−2^	Wang et al.^[^ ^99^ ^]^
Vacancies	Ru_0.85_Zn_0.15_O_2‐δ_	190	50 h@ 10 mA cm^−2^	Hou et al.^[^ ^100^ ^]^
	Rh‐RuO_2_	161	700 h@ 50 mA cm^−2^	Wang et al.^[^ ^62^ ^]^
	3R‐IrO_2_	188	500 h@ 10 mA cm^−2^	Fan et al.^[^ ^63^ ^]^
	Ag‐Co_3_O_4_(400)	470	/	Yan et al.^[^ ^65^ ^]^
	Mn_0.73_Ru_0.27_O_2‐δ_	208	10 h@ 10 mA cm^−2^	Wang et al.^[^ ^101^ ^]^
	NC@Vo·‐RuO_2_/CNTs‐350	170	>900 h@ 10 mA cm^−2^	Yan et al.^[^ ^22a^ ^]^
Edge	MEO‐GP	270	320 h@ 20 mA cm^−2^	Lu et al.^[^ ^68^ ^]^
	HCL	330	/	Sun et al.^[^ ^69^ ^]^
	Amorphous Ir NSs	255	8 h@ 10 mA cm^−2^	Wu et al.^[^ ^72^ ^]^
	RuO_2_ nanosheets	∼ 255	6 h@ 10 mA cm^−2^	Laha et al.^[^ ^71^ ^]^
	RuCu NSs	236	13.5 h@ 5 mA cm^−2^	Yao et al.^[^ ^73^ ^]^
	e‐H‐Na‐213	270	1300 h@ 10 mA cm^−2^	Wang et al.^[^ ^102^ ^]^
Heterostructure	Ir‐MoO_3_	∼156	48 h@ 100 mA cm^−2^	Liu et al.^[^ ^82^ ^]^
	Ni_3_S_2_@NiSe/NF	206(@50 mA cm^−2^)	36 h@ 10 mA cm^−2^	Shaikh et al.^[^ ^84^ ^]^
	Ru/RuS_2_	201	24 h@ 10 mA cm^−2^	Zhu et al.^[^ ^8^ ^]^
	RuO_2_‐WC NPs	347	10 h@ 10 mA cm^−2^	Sun et al.^[^ ^85^ ^]^
	Co_3_O_4_/CeO_2_	423	50 h@ 10 mA cm^−2^	Huang et al.^[^ ^86^ ^]^
	IrO_2_/GCN	276	4 h@ 20 mA cm^−2^	Chen et al.^[^ ^87^ ^]^
	IrO_2_@Ir/Co_3_O_4_	284	7 h@ 10 mA cm^−2^	Hou et al.^[^ ^89^ ^]^
	Ir/Nb_2_O_5‐x_	218	105 h@ 10 mA cm^−2^	Shi et al.^[^ ^88^ ^]^
	H/d‐MnO_x_/RuO_2_	178	40 h@ 10 mA cm^−2^	Wu et al.^[^ ^103^ ^]^
	Ir‐Sn PSC	193	260 h@ 20 mA cm^−2^	Zheng et al.^[^ ^104^ ^]^
	IrO_2_@TaB_2_	288	>120 h@ 10 mA cm^−2^	Wang et al.^[^ ^105^ ^]^
Defects induced strain	Li_0.52_RuO_2_	156	70 h@ 10 mA cm^−2^	Qin et al.^[^ ^92^ ^]^
	s‐RuO_2_/ATO	198	150 h@10 mA cm^−2^	Huang et al.^[^ ^20d^ ^]^
	E–IrO_2_/Ir	285	70 h@ 10 mA cm^−2^	Liu et al.^[^ ^66b^ ^]^
	Ru_1_–Pt_3_Cu	220	28 h@ 10 mA cm^−2^	Yao et al.^[^ ^44^ ^]^
	GB‐Ta_0.1_Tm_0.1_Ir_0.8_O_2−δ_	198	500 h@ 10 mA cm^−2^	Hao et al.^[^ ^51^ ^]^
	Ts‐Ir/MnO_2_	198	100 h@ 200 mA cm^−2^	Su et al.^[^ ^106^ ^]^
	Sn‐RuO_2_	184	150 h@ 10 mA cm^−2^	Xu et al.^[^ ^107^ ^]^
Defects induced amorphous structure	RuMn alloy	239	720 h@ 10 mA cm^−2^	An et al.^[^ ^93^ ^]^
	a‐RuTe_2_ PNRs	245	/	Wang et al.^[^ ^15b^ ^]^
	Li‐IrO_x_	300	10 h@ 10 mA cm^−2^	Gao et al.^[^ ^95^ ^]^
	Am‐Ir_1_Ru_3_O_8_ NBs	204	∼75 h@ 10 mA cm^−2^	Li et al.^[^ ^108^ ^]^

## 3 Phase Engineering

### 3.1 Binary Metal Oxides

**Figure 7:**
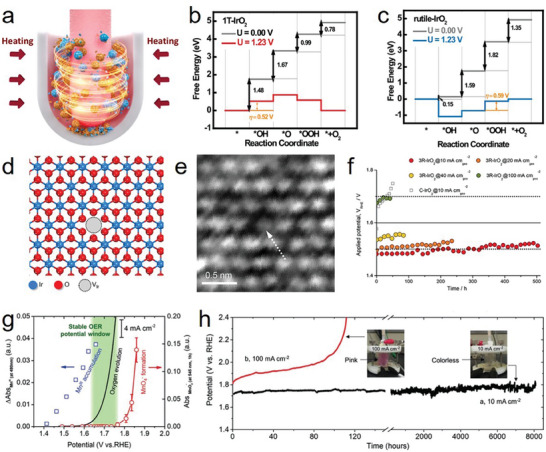
a) Schematic representation of the mechano‐thermal reactor for preparing 1T‐IrO_2_, where the mechanical and thermal operations are controlled simultaneously. The blue and yellow balls indicate IrCl_3_ and KOH, respectively. b) The free energy profile of OER over the 1T‐IrO_2_. c) The free energy profile of OER over rutile‐IrO_2_ (110) surface. a–c) Reproduced with permission.^[^ ^110^ ^]^ Copyright 2021, Springer Nature. d) High‐magnification STEM‐ADF image further shows the Ir vacancy defect. Reproduced with permission.^[^ ^63^ ^]^ Copyright 2021, Elsevier. e) UV/Vis absorption spectra of the electrolyte after 1 h of electrolysis at the indicated potentials. f) Potential dependence of Mn^3+^ accumulation (blue squares), the oxygen evolution current (black line), and MnO_4_^−^@ generation (red circles). e,f) Reproduced with permission.^[^ ^20b^ ^]^ Copyright 2019, Wiley‐VCH.

IrO_2_/RuO_2_ is the benchmark catalyst for the acidic OER. Further improvements in the catalytic activity and stability of different crystalline phases during the acidic OER have been examined.^[^ ^9^, ^109^ ^]^ Different crystal phases of IrO_2_/RuO_2_ exhibit higher catalytic performance than that of the benchmark rutile phase because of their unique atomic arrangement. Shao et al. employed a new strategy combining mechanochemistry and thermal treatment in a strongly alkaline environment to develop 1T‐IrO_2_ (**Figure** **7**a), which exhibited excellent acidic OER performance. They reported an overpotential of 197 mV at 10 mA cm^−2^ and high stability after 126 h of chronopotentiometry measurements at a high current density of 250 mA cm^−2^ in the PEM device.^[^ ^110^ ^]^ Theoretical calculations revealed that the free energy of *OH formation on Ir sites in 1T‐IrO_2_ was optimized (Figure 7b,c), thus improving the acidic OER performance. 1T‐IrO_2_ consisted of a two‐dimensional film with a thickness of 3–5 nm owing to its prolonged mechanochemical time, which increased the number of exposed active sites. However, the synthesis temperature of 1T‐IrO_2_ reached 800 °C, which was higher than that for conventional thermal synthesis. Subsequently, 3R‐IrO_2_ was prepared using a microwave‐assisted method instead of employing exceedingly high temperatures.^[^ ^63^ ^]^ In 3R‐phase IrO_2_, the unique active sites in the edge‐sharing octahedron were responsible for the enhanced performance, whereas the two‐dimensional thin film with abundant Ir vacancies further improved the proton and mass transport capacity (Figure 7d,e). Specifically, compared to commercial IrO_2_, this catalyst has an order of magnitude increase in stability (Figure 7f). Liao et al. developed a new monoclinic‐phase IrO_2_ nanoribbon catalyst, which exhibited excellent acidic OER performance because its d‐band energy level was lower than that of rutile IrO_2_, resulting in a weaker adsorption of O* during operation.^[^ ^111^ ^]^

Compared with noble metals, non‐noble metal oxides are unsatisfactory in terms of acid resistance and electric corrosion resistance; therefore, they are not readily applicable to the acidic OER. However, some phases of non‐noble metal oxides exhibit excellent performance and stability during acidic OER processes. Li et al. demonstrated that γ‐MnO_2_ was considerably stable in an acidic electrolyte (pH 0.2) for >8000 h in the OER potential window between 1.6 and 1.75 V (vs. RHE) (Figure 7g,h).^[^ ^20b^ ^]^ As confirmed by UV/vis spectra, the absorption peak of MnO_4_^−^ was detected when the potential exceeded 1.8 V vs RHE, indicating that γ‐MnO_2_ was irreversibly dissolved, thus decreasing the performance and even leading to the disintegration of γ‐MnO_2_. As the dissolution and redeposition of Mn^2+^ were balanced, γ‐MnO_2_ could remain in a stable potential window during the long‐term stability test.

Moreover, some common single‐metal spinel materials, such as Co_3_O_4_, demonstrate excellent catalytic performance; however, they cannot exist stably with a high oxidation potential. Therefore, the electronic structure must be further modified.^[^ ^65^ ^]^

### 3.2 Solid Solutions

**Figure 8:**
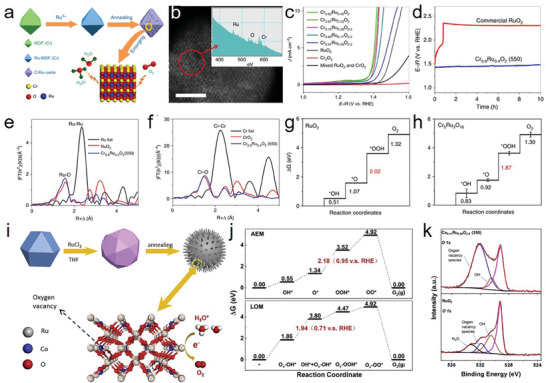
a) Schematic illustration of the preparation of Cr_0.6_Ru_0.4_O_2_ electrocatalysts. b) atomic‐resolution HAADF‐STEM images and EELS analysis (inset of b), scale bars: 5 nm. c) LSV results of Cr_x_Ru_1‐x_O_2‐δ_. d) Chronopotentiometry performance under a constant current density of 10 mA cm^−2^. e) Fourier transformed EXAFS spectra of Ru edge for Cr_0.6_Ru_0.4_O_2_(550), Ru foil, and commercial RuO_2_. f) Fourier transformed EXAFS spectra of Cr edge for Cr_0.6_Ru_0.4_O_2_(550), Cr foil, and commercial CrO_2_. g,h) The calculated free energy diagrams for RuO_2_ and Cr_5_Ru_3_O_16_. a–h) Reproduced with permission.^[^ ^113a^ ^]^ Copyright 2019, Springer Nature. i) Illustration of the synthesis of Co‐doped RuO_2_ nanorods. j) The free energy diagrams of the two mechanisms of LOM and AEM. The rate‐determining barriers together with that versus RHE are denoted. k) O 1s XPS profiles of Co_0.11_Ru_0.89_O_2‐δ_ (350) and RuO_2_. i–k) Reproduced with permission.^[^ ^23b^ ^]^ Copyright 2020, Elsevier.

Compared with the development of new phase materials, enhancing the activity and stability of basic phase materials remains a crucial area of research. Solid solutions, typically single‐phase metals or oxide alloys, are suitable candidates in this regard. These materials can be used to adjust the electronic and interfacial structures of the active species through the introduction of additional metal species, thereby optimizing the binding energy between the active centers and reaction intermediates. This strategic adjustment accelerates the kinetic rate; consequently, solid solutions can effectively enhance the performance of basic phase catalysts. Doping non‐precious metals such as W,^[^ ^38^ ^]^ Co,^[^ ^23^, ^59^, ^112^ ^]^ Mn,^[^ ^23^, ^101^ ^]^ Cr,^[^ ^113^ ^]^ Ni,^[^ ^114^ ^]^ Cu,^[^ ^26b^ ^]^ Zn,^[^ ^115^ ^]^ In,^[^ ^116^ ^]^ and Fe^[^ ^30^ ^]^ into IrO_2_ or RuO_2_ lattices can successfully regulate the electronic structure of the active sites and markedly enhance the OER performance in acidic media. Metallic cation exchange with metal–organic frameworks (MOFs) followed by post‐treatment provides a simple and easily controllable strategy to obtain solid solutions. The resulting solid solution has an optimal electronic structure and can maintain the basic structure of the MOF at the microscale, which improves the performance of the catalyst beyond that of bulk materials. Lin et al. reported a series of Cr_x_Ru_1−x_O_2_ (T) (0 < x < 1; T is the treatment temperature (°C)) solid‐solution catalysts with uniform element distribution via Ru^3+^ exchange with MIL‐101(Cr) in tetrahydrofuran solution (**Figure** 8a).^[^ ^113a^ ^]^ In this study, they confirmed that the presence of Cr was the primary reason for the improvement in the OER performance in 0.5 M H_2_SO_4_; moreover, the substantially enhanced catalyst stability was predominantly ascribed to the low occupation at the Fermi level. Based on the atomic‐resolution high‐angle annular dark‐field scanning transmission electron microscopy images and electron energy loss spectra of Cr_0.6_Ru_0.4_O_2_ (550), the observation of a uniformly crystallized single nanocrystal (Figure 8b) confirmed that Ru and Cr atoms coexisted in a uniform distribution. Moreover, Cr_0.6_Ru_0.4_O_2_ (550) demonstrated higher performance and stability than the solid solutions with other molar ratios (Figure 8c,d). In Cr_0.6_Ru_0.4_O_2_ (550), the Cr–O length was slightly elongated from 1.47 (RuO_2_) to 1.50 Å, and the length of Ru–O was slightly shortened. This confirmed that the fine structure of Ru changed (Figure 8e,f). In addition, considering the calculated free energy diagrams, the free energy change in the RDS at the Ru sites on the Cr_5_Ru_3_O_16_ surface (1.87 eV) was ≈0.1 eV lower than that on the RuO_2_ surface (2.02 eV), which was consistent with the decreased overpotential of ∼100 mV, as measured in the experiments (Figure 8g,h). Similarly, some mixed heteroatom solid solution oxides such as W_0.99_Ir_0.01_O_3−δ_ (1 wt%), Ir_0.7_Co_0.3_O_x_, and Mn_0.73_Ru_0.27_O_2−δ_ have been synthesized.^[^ ^101^, ^117^ ^]^ However, solid solutions formed by doping with heteroatoms typically cause vacancies, strains, and other defects owing to the size difference between the atoms, which is inevitable. Zhang et al. demonstrated that Cr leaching and strong Cr–Ir interactions resulted in high‐chemical‐state oxides of Ir, which exhibited superior activity for the acidic OER.^[^ ^113b^ ^]^ Shi et al. found that the reaction route, and subsequently the stability, could be customized by modulating the Ru charge via the formation of a MRuO_x_ (M = Ce^4+^, Sn^4+^, Ru^4+^, and Cr^4+^) solid solution.^[^ ^10^ ^]^ This study indicated that the Ru charge could be used to regulate the reaction path of the catalyst, as verified by the charge redistribution in Ru–O–M resulting from the different ionic electronegativities. Furthermore, oxygen vacancies were substantially increased during the electrochemical process, and the synergy between the abovementioned factors promoted the OER activity in acidic media.

Specific active centers must be distinguished, and the origin of their activity must be examined. Tian et al. reported that transition metal atoms could modify the electronic structure of RuO_2_ and provide abundant oxygen vacancies through the different properties between heteroatoms (Figure 8i‐k), which increased the OER performance in acidic media.^[^ ^23b^ ^]^ Furthermore, the formation of Ru(OH)–O_v_(OH) after the second attack of H_2_O was identified as RDS, which presented a barrier of 0.71 eV (vs. RHE) for LOM, which was lower than that for AEM (0.95 eV vs RHE) (Figure 8i). The decrease in the energy barrier (0.24 eV) implied that the RDS of the OER preferentially occurred on the O vacancies rather than on the Ru sites. The existence of O vacancies further improved the stability of RuO_2_, effectively preventing the overoxidation of Ru to form soluble RuO_4_ species. Moreover, strain significantly affected the crystal phase and the catalyst–intermediate interaction, which strongly influenced the behavior of the intermediate at the active site. Qin et al. demonstrated that the Li‐induced strain reconstructed the surface of the Li_x_RuO_2_ solid solution, and the dangling O atoms near Ru sites served as proton acceptors, thus regulating the binding energy of the catalyst intermediates.^[^ ^92^ ^]^

Modifying the electronic structure of the active center by doping elements in the solid solution can enhance the catalytic performance, and further analyzing the role of the doped elements generally yields unexpected results. For example, many previous studies have indicated that the deprotonation step of protonated bridged oxygen (HO_bri_) accelerates the kinetics of the acidic OER, and it is considered to be the RDS.^[^ ^15^, ^38^, ^118^ ^]^ Wen et al. synthesized a solid solution of Ru_5_W_1_O_x_ with rutile phase, which exhibited a low overpotential (235 mV@ 10 mA cm^−2^) and degradation rate (0.014 mV h^−1^) over 550 h, based on the chronopotentiometry results.^[^ ^38^ ^]^ W was atomically dispersed in the lattice of RuO_2_ and demonstrated strong Brønsted acidity through the formation of W–O_bri_–Ru sites, which reduced the high proton adsorption energy of O_bri_ on RuO_2_ and facilitated proton transfer from oxo‐intermediates to the neighboring O_bri_; finally, the overall acidic OER kinetics were accelerated.

Recently, high entropy alloys (HEAs) have attracted much attention due to their huge multi‐element composition space and unique high entropy mixed structure. Compared with pure metals or low‐element alloys, HEAs can achieve high activity, high selectivity, improved stability and reduced cost through flexible component design and various element regulation.^[^ ^119^ ^]^ Therefore, HEAs are widely used in electrocatalysis, which is of great significance for the industrial sustainable development of clean energy. The unique properties of HEAs (high entropy, lattice distortion, hysteresis diffusion and cocktail effect) further enhance the catalytic performance of Ir‐/Ru‐based materials for acidic OER.^[^ ^120^ ^]^

Firstly, high entropy materials utilize relatively inexpensive metals, significantly reducing costs.^[^ ^121^ ^]^ For instance, IrFeCoNiCu‐HEA NPs, IrRuNiMoCo HEA, and ZnNiCoIrMn HEA with low‐Ir/Ru content have been developed for efficient acidic OER.^[^ ^120^, ^122^ ^]^ In these studies, scarce noble metals are largely replaced by abundant transition metals, thereby reducing costs. Furthermore, a more stable and active Ir/Ru shell is formed through the dissolution of partially transition metals. Yao et al. reported that core–shell structure catalyst of IrRuNiMoCo HEA coated by Ir‐rich IrRuNiMo medium‐entropy oxide (HEA@Ir‐MEO) exhibited excellent activity of 1.8 V/3.0 A cm^−2^@80 °C and long‐term stability with over 500 h@ 1.0 A cm^−2^ in a PEM device. The source of the superior performance of HEA@Ir‐MEO is that the Ir‐rich MEO shell inhibits the structure evolution during the acidic OER process.^[^ ^122^ ^]^ It is noteworthy that the electronic structure of the Ir‐rich surface can be tuned by the HEA core with different compositions, affecting the adsorption energy of intermediates and further optimizing the energy barrier.^[^ ^123^ ^]^

In addition to the acid‐resistant shell that protects the HEA core, enhancing durability, HEAs inherently stabilize themselves through flexible electronic structure regulation and controllable element combinations. The high entropy and cocktail effects confer superior anti‐oxidation and anti‐corrosion properties, even under harsh conditions, thereby extending the catalyst's lifespan. For example, Tajuddin et al. developed a non‐noble metal HEA incorporating elements such as Ti, Zr, Nb, and Mo for passivation to improve stability, and Cr, Ni, Co, Fe, and Mn to enhance activity for acidic OER.^[^ ^124^ ^]^ This HEA (9 elements) demonstrated excellent activity and stability during the CV tests, addressing the challenge of renewable energy fluctuation. Replacing scarce Ir/Ru noble metals with more abundant transition metals in the anode electrodes of PEMWEs is crucial for cost reduction and advancing hydrogen energy development. The ongoing development of low‐content noble metals or even noble‐free HEAs, combining transition metals with high entropy, presents a promising approach.

Moreover, the synergistic effect of multiple elements in high‐entropy materials, and various intrinsic defects can optimize the electronic structure and reaction interface characteristics of the active center, thereby customizing catalysts suitable for different water electrolysis conditions and requirements. This design flexibility also provides broad space for the development of efficient and durable acidic OER catalysts. Yu et al. demonstrated that the intrinsic nature of high conformational entropy stabilizes the involvement of lattice O during the catalysis, which was proved by isotopic gas detection and electron spin resonance.^[^ ^125^ ^]^ Besides, the regulation of inherent defects in HEAs can significantly enhance their catalytic performance. For example, Hu and co‐workers proved that the synergistic effects between multiple foreign metal elements and grain boundaries (GBs) can optimize the binding energy of catalysts with O‐intermediates, thus causing enhanced performance with over a 500‐h durability test in PEMWEs.^[^ ^126^ ^]^

These superior HEAs for acidic OER not only meet the performance requirements of devices, but further reduce the dosage of noble metals, which is consistent with the challenges of future industrial costs. Therefore, it is of great practical significance to design suitable HEAs with low‐cost, high activity, and long‐term stability for acidic OER. Finally, high‐entropy materials reduce the reliance on rare and limited precious metals in raw materials, which contributes to sustainable development and environmental protection, as well as being in line with the principle of green hydrogen production.

### 3.3 Polynary Metal Oxides: Pyrochlore, Perovskite, and Spinel Oxides

Some metal oxides, such as pyrochlore, perovskite, and spinel oxides, have highly hybridized metallic d and O 2p orbitals, which optimize the electronic structure of the metal centers.^[^ ^127^ ^]^ Hence, new‐phase Ir‐ and Ru‐based oxides have recently received increasing attention.

**Figure 9:**
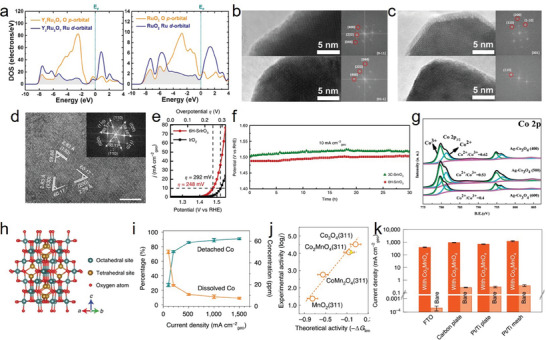
a) Calculated PDOS plots of Ru 4d and O 2p orbitals for Y_2_Ru_2_O_7_ and RuO_2_. Shaded area shows the overlapped bands between Ru 4d and O 2p orbitals. The Fermi level is set to zero. b,c) HRTEM micrographs (left) and FFT images of near‐surface region (right), showing the crystalline surface structure of Y_2_Ru_2_O_7−δ_ while amorphous surface layer formed on RuO_2_ catalysts after the cycle tests. a–c) Reproduced with permission.^[^ ^129^ ^]^ Copyright 2017, ACS Publications. d) HRTEM image of 6H‐SrIrO_3_. Scale bar, 5 nm. Inset: the corresponding fast Fourier transform image. e) Polarization curves of 6H‐SrIrO_3_ and IrO_2_ in 0.5 m H_2_SO_4_ solution with 85% iR‐compensations. The current densities are normalized by the geometric area. f) Chronopotentiometric curves for OER in the presence of 6H‐SrIrO_3_ and 3C‐SrIrO_3_ in 0.5 m H_2_SO_4_ solution at 10 mA cm^−2^_geo_ (without iR compensations). d–f) Reproduced with permission.^[^ ^133^ ^]^ Copyright 2018, Springer Nature. g) XPS spectra Co 2p of Ag‐Co_3_O_4_(400), Ag‐Co_3_O_4_(500) and Ag‐Co_3_O_4_(600). Reproduced with permission.^[^ ^65^ ^]^ Copyright 2018, Royal Society of Chemistry. h) Crystal structure of Co_2_MnO_4_. i) The percentage and concentration of Co that was mechanically detached in solid form (green) or dissolved from the catalyst (brown), after electrolysis in H_2_SO_4_ (pH 1). The *x*‐axis indicates the current density at which electrolysis was performed, and quantification was performed using ICP‐MS after the catalyst was fully deactivated. Data points with error bars were obtained by the standard deviation of at least three independent measurements. j) The correlation between experimental activities (log *j*) and theoretical ones (–Δ G_lim_) derived from the Δ*G*‐limiting energies. A 0.2 eV error bar is shown to describe the theoretical uncertainty and includes the solvation correction, functional with Hubbard U calculations, and system error from DFT calculations. The orange circles indicate the theoretical data based on perfect surfaces, and the yellow‐filled triangle shows the ΔG‐limiting energy of Co_2_MnO_4_ as an average with the consideration of all the possible structures. (k) Current densities of Co_2_MnO_4_ at 1.8 V vs RHE after iR correction. The activities of the bare substrates are also shown for comparison. Data points with error bars were obtained by the standard deviation of at least three independent measurements. h–k) Reproduced with permission.^[^ ^20a^ ^]^ Copyright 2021, Springer Nature.

Because of their universal and adjustable structure, pyrochlore oxides are generally formulated as A_2_B_2_O_6_O’, where eight‐coordinated A‐sites are occupied by alkaline metals and six‐coordinated B‐sites are typically occupied by Ir and Ru. Pyrochlore oxides such as Y_2_Ir_2_O_7_, Bi_2_Ir_2_O_7_, and Pb_2_Ir_2_O_6.5_ have recently been discovered and applied to the acidic OER.^[^ ^128^ ^]^ Yang et al. developed a pyrochlore‐type Y_2_Ru_2_O_7−δ_ using the sol–gel method.^[^ ^129^ ^]^ In this study, the overlap between Ru 4d and O 2p orbitals lowered the band center energy, which provided a more stable Ru–O bond in Y_2_Ru_2_O_7−δ_ than that in pure RuO_2_. The electronic band structures between Y_2_Ru_2_O_7−δ_ and RuO_2_ were significantly different (**Figure** **9**a). The band center energy of Y_2_Ru_2_O_7−δ_, between Ru 4d and O 2p orbitals, was away from the Fermi level, which enhanced the stability. This finding was consistent with the HRTEM micrographs and fast Fourier transform images before and after the cycle tests; the surface of pure RuO_2_ turned from crystalline to amorphous; however, Y_2_Ru_2_O_7−δ_ remained intact (Figure 9b,c). Moreover, the large radii of the A‐site atoms with weaker electronic correlations promoted higher OER activity in acidic media to some extent.^[^ ^130^ ^]^ The substitution of metal cations at the A or B sites can further improve the performance. Other elements can also play an important role in modifying the electronic structure: for example, Bi_x_Er_2−x_Ru_2_O_7_, Y_2_[Ru_1.6_Y_0.4_]O_7−σ_, Pb_2_[Ru_2−x_Pb_x_]O_7−δ_, and (Na_0.33_Ce_0.67_)_2_(Ir_1−x_Ru_x_)_2_O_7_.^[^ ^131^ ^]^

Perovskite oxides (ABO_3_), wherein A‐sites are occupied by various rare‐earth/alkaline metals and B‐sites are occupied by transition metals, have attracted considerable attention because their structure is modified by cationic substitutions with various metal species, regardless of the valence states at the A/B sites.^[^ ^132^ ^]^ Such highly variable and adjustable structures and properties enable perovskites to be more advantageous than other emerging materials. Zou et al. developed Ir‐based perovskites with reduced noble metal content.^[^ ^29^, ^133^ ^]^ Corrosion‐resistant and highly efficient 6H‐SrIrO_3_ perovskite with lower Ir content (27% less than IrO_2_) was synthesized, and it exhibited excellent acidic OER performance with a low overpotential of 248 mV at 10 mA cm^−2^ and high stability for 30 h (Figure 9d–f).^[^ ^133^ ^]^ Subsequently, Zou et al. investigated cation exchange to synthesize low‐noble‐content catalysts, such as SrTi_0.67_Ir_0.33_O_3_ (57% less than IrO_2_).^[^ ^29^ ^]^ In a subsequent study, Chen et al. developed two‐dimensional perovskite Sr_2_IrO_4_ NSs (HION), which exhibited satisfactory OER performance with an overpotential of 300 mV at 10 mA cm^−2^ and stability for 30 h in 0.1 M HClO_4_.^[^ ^134^ ^]^ Ir leaching from HION was considerably lower than that from SrIrO_3_ and IrO_2_ during 10 h of electrocatalysis. Moreover, Ir/Ru‐based perovskites are not limited to being used as corrosion‐resistant and stable catalysts; studies have also shown that the metal cations in perovskites dissolve to reconstruct the active center during the catalytic process, which can achieve the expected purpose.^[^ ^60^ ^]^

In addition to pyrochlore and perovskite oxides, spinel oxides are acidic OER catalysts that have been recently studied. Uniform spinel oxides composed of a single metal species maintain a certain level of stability in acidic electrolytes; however, they dissolve rapidly when a high oxidation potential is applied. To address this problem, several methods have been developed. Yang et al. reported that spinel cobalt oxides synthesized by hydrothermal treatment at different temperatures have different ratios of Co^2+^/Co^3+^, which severely affect their long‐term stability.^[^ ^64^ ^]^ This theory has been accepted by some researchers (Figure 9g).^[^ ^65^ ^]^ However, some researchers consider the stability to be predominantly affected by the metal species occupying the octahedral sites. Li et al. synthesized spinel Co_2_MnO_4_, wherein the octahedral sites were occupied by Mn^4+^/Mn^3+^ together with Co^3+^, whereas Co^2+^ tended to occupy the tetrahedral sites (Figure 9h).^[^ ^20a^ ^]^ The stronger Mn–O bonds markedly improved the stability of Co_2_MnO_4_ compared with that of spinel Co_3_O_4_ and prevented the dissolution of O_1_ (Figure 9i). This study also revealed that the theoretical and experimental activity of Co_2_MnO_4_ approached that of Co_3_O_4_ (Figure 9j), with excellent acidic OER performance (Figure 9k); however, it was considerably more stable than Co_3_O_4_, and it could be operated for 1500 h at a current density of 200 mA cm^−2^_geo_ under extreme conditions. Similarly, Anantharaj et al. reported that Co_2_TiO_4_ exhibited excellent performance, which was comparable to that of IrO_2_ and higher than that of Co_3_O_4_ in an identical experimental test.^[^ ^135^ ^]^ Research on spinel materials used as acidic OER catalysts is in its initial stages, and various problems are to be solved, such as the stable hydrous oxide layer on the surface of spinel oxides under acidic electrolytes and the balance between the vacancies and activity/stability (**Table** **2**).^[^ ^64^, ^65^ ^]^

**Table 2:** Typical catalysts of Phase Engineering for acidic OER.

Phase engineering	Catalysts	Overpotential [mV] at 10 mA cm^−2^	Stability	Reference
Binary metal oxides	1T‐IrO_2_	197	45 h@ 50 mA cm^−2^	Dang et al.^[^ ^110^ ^]^
	3R‐IrO_2_	188	511 h@ 10 mA cm^−2^	Fan et al.^[^ ^63^ ^]^
	γ‐MnO_2_	489±5	8000 h@ 10 mA cm^−2^ (pH 2)	Li et al.^[^ ^20b^ ^]^
Solid solution	Cr_0.6_Ru_0.4_O_2_	178	10 h@ 10 mA cm^−2^	Lin et al.^[^ ^113a^ ^]^
	W_0.99_Ir_0.01_O_3−δ_	500±26	2000 s@ 10 mA cm^−2^	Kumari et al.^[^ ^117b^ ^]^
	In_0.17_Ru_0.83_O_2_‐350	177	20 h@ 10 mA cm^−2^	Chen et al.^[^ ^116^ ^]^
	Co‐RuO_2_ nanorods	169	50 h@ 10 mA cm^−2^	Tian et al.^[^ ^23b^ ^]^
	Li_0.52_RuO_2_	156	70 h@ 10 mA cm^−2^	Qin et al.^[^ ^92^ ^]^
	Ru_5_W_1_O_x_	235	550 h@ 10 mA cm^−2^	Wen et al.^[^ ^38^ ^]^
	SnRuO_x_	194	250 h@ 100 mA cm^−2^	Shi et al.^[^ ^10^ ^]^
	Ir_0.3_Cr_0.7_O_2_	255	200 h@ 10 mA cm^−2^	Zhang et al.^[^ ^113b^ ^]^
	Mn_1_Co_5_O_x_	275	∼300 h@ 100 mA cm^−2^	Zhang et al.^[^ ^136^ ^]^
	Re_0.06_Ru_0.94_O_2_	190	200 h@ 10 mA cm^−2^	Jin et al.^[^ ^137^ ^]^
	RuNiMoCrFeO_x_/CNT	219	100 h@ 100 mA cm^−2^	Yu et al.^[^ ^125^ ^]^
	M‐RuIrFeCoNiO_2_	189	120 h@ 10 mA cm^−2^	Hu et al.^[^ ^126^ ^]^
	IrFeCoNiCu‐HEA NPs	∼302	12 h@ 10 mA cm^−2^	Maulana et al.^[^ ^120b^ ^]^
	CoFeNiMoWTe	373	100 h@ 10 mA cm^−2^	Jo et al.^[^ ^138^ ^]^
	(Ru_0.2_Ir_0.2_Cr_0.2_W_0.2_Cu_0.2_)O_2_	220	12 h@ 10 mA cm^−2^	Miao et al.^[^ ^139^ ^]^
	ZnNiCoIrMn	237	100 h@ 10 mA cm^−2^	Kwon et al.^[^ ^120a^ ^]^
Polynary metal oxides	Y_2_Ir_2_O_7_	/	24 h@ 10 mA cm^−2^	Shih et al.^[^ ^128a^ ^]^
	Y_2_Ru_2_O_7−δ_	/	8 h@ 1 mA cm^−2^	Kim et al.^[^ ^129^ ^]^
	Bi_2_Ir_2_O_7_	/	/	Lebedev et al.^[^ ^128b^ ^]^
	6H‐SrIrO_3_	248	30 h@ 10 mA cm^−2^	Yang et al.^[^ ^133^ ^]^
	Sr_2_IrO_4_ NSs (HION)	300	30 h@ 10 mA cm^−2^	Chen et al.^[^ ^134^ ^]^
	Co_2_MnO_4_	298	1500 h@ 200 mA cm^−2^	Li et al.^[^ ^20a^ ^]^
	Co_2_TiO_4_	513	/	Anantharaj et al.^[^ ^135^ ^]^

## 4 Structure Engineering

Applications of Ru‐ and Ir‐based oxides, regarded as state‐of‐the‐art catalysts for the acidic OER, are hindered by the limited availability of noble metals in the Earth's crust. Consequently, researchers must focus on enhancing the service life of these catalysts.^[^ ^9b^ ^]^ The structure of catalytically active noble metals and certain active transition metal oxides must be optimized to enhance the activity and stability. Particularly, controlling defects and generating new active phases at the atomic scale, along with well‐designed nanoscale structures that enhance the exposure of active sites, can improve the catalyst performance.

Nanostructure modulation is a targeted approach to address issues related to catalytic efficiency and service life owing to the inherent structure of catalysts. For example, the implementation of a core–shell structure serves as an effective strategy to protect vulnerable active centers; this strategy was originally proposed to extend the service life of the catalysts.^[^ ^140^ ^]^ In addition, the fabrication of porous and low‐dimensional materials has emerged as a direct and highly effective method to enhance the catalytic efficiency. The performance of these materials is superior to that of bulk materials because of their large exposed specific surface areas and increased number of active centers. Nanostructures provide numerous benefits for PEMWEs, including a high specific surface area that enhances catalytic activity, core–shell structures that improve durability, porous and low‐dimensional designs that reduce loading requirements and enhance mass transport, and self‐supported configurations that eliminate the need for binders. These advantages are highly beneficial for industrial applications, where they address the challenges faced by PEMWEs.

However, modifying the electronic structure enables certain secondary materials to emulate benchmark catalysts, thus increasing their significance in practical applications. For example, transition metal carbides demonstrate a Pt‐like electronic structure, and their catalytic performance is comparable to that of Pt‐based catalysts to some extent; consequently, they are promising alternatives to Pt‐based catalysts.^[^ ^141^ ^]^ Furthermore, by binding with other heteroatoms (as observed in solid solutions^[^ ^101^ ^]^ and ADCs^[^ ^40^ ^]^), the electronic structure can be optimized to better satisfy the requirements of catalytic processes.

Nanostructure modulation is frequently accompanied by the tuning of the electronic structure. In this review, only a brief introduction to this topic has been provided.

### 4.1 Core–Shell

**Figure 10:**
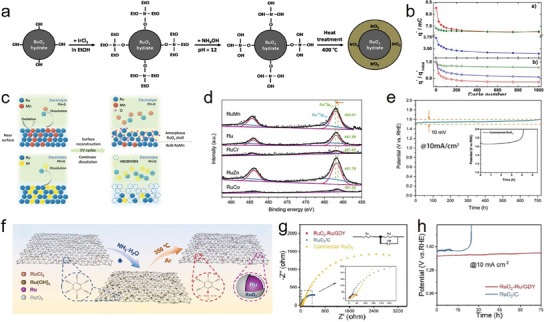
a) Synthesis Procedure of the Iridium (IV) Oxide Coated on Commercial Ruthenium (IV) Oxide Nanoparticles. b) Charge (q*) evolution (a) and q*/q* initial ratio (b) for RuO_2_ after heat treatment (blue), IrO_2_ (red), and IrO_2_ @RuO_2_ catalyst (green) electrocatalysts during stability test measurement. a,b) Reproduced with permission.^[^ ^142c^ ^]^ Copyright 2016, ACS Publications. c) Schematic of surface reconstruction for RuMn and dissolution, for unstable Ru‐based alloys in acidic media during CV cycles. d) XPS spectra of pristine Ru, RuCr, RuCo, RuZn, and RuMn. e) The chronopotentiometry of RuMn and commercial RuO_2_ at 10 mA cm^−2^. (c‐e) Reproduced with permission.^[^ ^93^ ^]^ Copyright 2022, Wiley‐VCH. f) Schematic illustration of the synthetic route for RuO_2_‐Ru/GDY. RuCl_3_/GDY (left). Ru(OH)_x_/GDY (mid). RuO_2_‐Ru/GDY (right). g) electrochemical impedance spectroscopy (EIS) of RuO_2_‐Ru/GDY, RuO_2_/C, and commercial RuO_2_. h) Chronopotentiometric curves of RuO_2_‐Ru/GDY and RuO_2_/C at a constant current density of 10 mA cm^−2^. f–h) Reproduced with permission.^[^ ^142a^ ^]^ Copyright 2022, IOP Publications.

For the acidic OER, the active centers are commonly coated with a corrosion‐resistant and durable shell to prolong their service life.^[^ ^142^ ^]^ A core–shell IrO_2_@RuO_2_ nanocatalyst was synthesized via precipitation (**Figure** **10**a), with the high activity of RuO_2_ and stability of IrO_2_, which substantially enhanced the OER performance and extended the lifetime of the catalyst in 0.5 M H_2_SO_4_.^[^ ^142c^ ^]^ The activity loss of IrO_2_@RuO_2_ (green line; Figure 10b) was found to be only 3.3%, considerably lower than that of IrO_2_ (12.2%; red line) and RuO_2_ (9.2%, blue line) after 1000 cycling tests; the use of the core–shell structure is an effective and simple strategy to prevent the leaching of the active centers. Carbon‐based materials are corrosion‐resistant and stable, with the economical and practical advantages of being low‐cost, environmentally friendly, non‐toxic, and easily available; consequently, they are suitable for electrocatalytic operations, particularly in harsh chemical environments.^[^ ^2^, ^143^ ^]^ Similarly, Liu et al. developed Si–RuO_x_@C complex NPs from 72 aromatic ring‐caged precursors; they were stable for >100 h of operation in acidic media owing to the carbon cage coating.^[^ ^34^ ^]^ Cui et al. reported that robust interface Ru centers between the RuO_2_ core and graphene shell considerably enhanced both the activity (227 mV at 10 mA cm^−2^) and stability (over 24 h), which were higher than those of commercial RuO_2_.^[^ ^144^ ^]^ Notably, the outer graphene shell changed a portion of the electronic structure of the RuO_2_ core, accordingly changing the activity and stability. Moreover, Ru centers were found to disrupt the conventional scaling relationship between the free energies of HOO* and HO*, thus decreasing the overpotential and increasing the intrinsic acidic OER activity. In addition to carbon‐based materials, some non‐noble metal oxides with similar characteristics, for example, TiO_2_^[^ ^145^ ^]^ and Co_3_O_4_,^[^ ^146^ ^]^ have been used in the acidic OER. Krivina et al. developed a composite catalyst consisting of IrO_x_ coated with an acid‐stable TiO_x_ layer via atomic‐layer deposition.^[^ ^24a^ ^]^ The acid‐stable TiO_x_ shell effectively prevented dissolution of the active Ir sites.

The volcano map^[^ ^147^ ^]^ reveals many earth‐abundant and highly active non‐noble metallic species such as Co‐ and Mn‐based materials, which are potential candidates for acidic OER catalysts.^[^ ^20^, ^65^, ^101^ ^]^ However, non‐noble metal species are considerably less stable than noble metals. Therefore, several researchers aimed to synthesize an appropriate structure to stabilize these non‐noble metals during the acidic OER. Yang et al. reported that a carbon‐coated Co_3_O_4_ composite catalyst derived from glucose exhibited excellent catalytic performance with a lifetime of >80 h.^[^ ^148^ ^]^ A similar study was recently conducted by Yu et al., considering a ZIF‐9‐derived Co_3_O_4_@C active center; graphite and paraffin oil were added as conductive and partially hydrophobic materials to provide dispersion and support.^[^ ^149^ ^]^ Moreover, Fe doping resulted in smaller primary particle sizes and a suitably optimized electronic structure of Co_3_O_4_.^[^ ^24b^ ^]^ In addition to using a durable shell to protect and stabilize Co_3_O_4_ to catalyze the acidic OER, reducing the Co^3+^ content or reducing the ratio of Co^3+^/Co^2+^ can achieve similar results.^[^ ^64^, ^65^ ^]^ Yeh et al. observed that core–shell FTO@Co_3_O_4_ NPs effectively inhibited the dissolution of Co_3_O_4_ while dispersing Co_3_O_4_. This is beneficial for a stable acidic OER.^[^ ^150^ ^]^ Furthermore, Co^3+^‐lean and oxygen vacancy‐free materials play crucial roles in the acidic OER, as well as in the FTO (F‐doped tin oxide) core to provide a conductive and acid‐resistant substrate. Therefore, the structural design can affect the electronic structure of the active center to match the catalytic reaction.

In addition to the formation of core–shell structures with different species, similar results were obtained via the phase transition of the active center itself. Chen et al. prepared an ultrasmall quasi‐core–shell Ru–RuO_2_ nanostructure.^[^ ^142a^ ^]^ Initially, the Ru species settled onto the graphdiyne (GDY) support to form Ru(OH)_x_/GDY in an alkaline environment; subsequently, Ru(OH)_x_ was partially reduced during air annealing, and a quasi‐core–shell Ru–RuO_2_/GDY nanostructure was formed (Figure 10f). The electronic structure of the RuO_2_ shell was regulated by the Ru core during its formation, which enabled the catalyst to exist more stably in a strong acid‐corrosive environment and at a high oxidation potential. Because of the presence of GDY, the conductivity of the catalyst and the dispersion of the Ru center were markedly enhanced (Figure 10g). However, it is still indispensable to the redox reactions of carbon materials at high temperatures. An et al. employed the opposite approach and obtained similar results.^[^ ^93^ ^]^ Amorphous RuO_x_ shells were formed on the RuMn alloy during the CV cycles, and the electronic structure of Ru was optimized, which is beneficial for long‐term operation (Figure 10c,d). This shell could still be detected after the ultralong lifetime test, thus highlighting the advantages of the core–shell structure and the excellent surface reconstruction strategy. The core–shell structures formed using the two methods with opposite synthesis paths were more stable than the original active center (Figure 10e,h). Both the low‐valence core formed by self‐reduction and high‐valence shell formed by self‐evolution and oxidation changed the properties of the active center, further improving the catalytic stability.

From the perspective of structure engineering, some problems have been solved at the surface level; however, more comprehensive solutions are required. The original index of the materials must be fundamentally improved. Therefore, the catalytic performance can be improved by adjusting the surface and/or electronic structure of the active centers (and catalysts) and reducing the reaction energy barriers and adsorption energies of OER intermediates. These strategies have been widely recognized by researchers and are being further investigated. With further research, the role of core–shell structure can gradually expand from protecting the vulnerable active centers to interactions between the core and shell materials; thus, electronic tuning can be achieved, and suitable catalysts can be employed for various catalytic reactions.

Furthermore, the electrocatalysts featuring a core–shell architecture provide numerous advantages for PEMWEs. In addition to the widely acknowledged benefits of improved catalytic activity and enhanced stability, the composition, size, and morphology of such catalysts can be meticulously tailored to optimize performance across diverse applications within PEMWE systems. This versatility underscores the core–shell structure as a promising avenue for the development of both efficient and durable catalysts, positioning them as viable candidates for future commercial implementations of PEMWE technology.

### 4.2 Porous Materials

As revealed by electrocatalytic studies, the development of high‐current‐density catalysts is necessary for practical applications. When the catalyst is in operation, bubbles arising on the catalyst surface should not affect the next step or even physically damage the catalyst; these issues must be addressed. In addition, many studies have revealed that the appearance and desorption of bubbles lead to jumps in the potential, which hinder the accurate description of the catalytic performance.^[^ ^151^ ^]^ Porous structures have been regarded as the most reliable choices. Catalysts with high current densities have a common feature, that is, a porous structure, which is conducive to electrolyte diffusion and the exposure of active centers.^[^ ^116^, ^152^ ^]^ Materials with numerous pores and large specific areas expose additional active sites, which can facilitate mass transport and charge exchange during catalysis.^[^ ^153^ ^]^ Herein, the preparation methods of porous materials are not extensively discussed; the focus is on the utilization of porous structures for the acidic OER.

**Figure 11:**
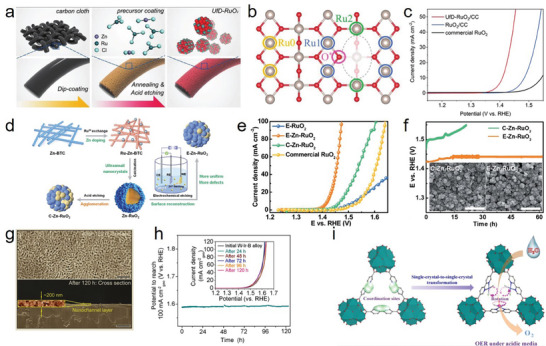
a) Illustration of the synthetic route for the UfD‐RuO_2_/CC. b) Top view of defective RuO_2_ structure. Gray and red spheres present Ru and O atoms, respectively. c) Polarization curves of UfD‐RuO_2_/CC, RuO_2_/CC, and commercial RuO_2_/CC after capacitance‐correction and iR‐correction. a–c) Reproduced with permission.^[^ ^22b^ ^]^ Copyright 2019, Wiley‐VCH. d) Illustration of the synthetic route for the E‐Zn‐RuO_2_ and C‐Zn‐RuO_2_. e) LSV polarization curves of E‐RuO_2_ (RuO_2_), E‐Zn‐RuO_2_, C‐Zn‐RuO_2_, and commercial RuO_2_. f) Stability of C‐Zn‐RuO_2_ and E‐Zn‐RuO_2_ at 10 mA cm^−2^; the insets are the morphologies of the two samples after the OER. d–f) Reproduced with permission.^[^ ^115^ ^]^ Copyright 2022, Royal Society of Chemistry. g) Surface and cross‐sectional SEM images of the W‐Ir‐B alloy catalyst after the 120 h acidic OER test. The inset shows the magnified cross‐section image of the nanochannel structure (yellow solid box). h) Chronopotentiometry curves of the W‐Ir‐B alloy catalyst in 0.5 m H_2_SO_4_ electrolyte at a current density of 100 mA cm^−2^_geo_ for 120 h. The inset shows the LSV curves taken periodically during the test (every 24 h). g,h) Reproduced with permission.^[^ ^152^ ^]^ Copyright 2021, Springer Nature. i) Schematic illustration of Th‐MOF electrocatalysis. Reproduced with permission.^[^ ^155^ ^]^ Copyright 2022, ACS Publications.

Chemical etching is a conventional method for the synthesis of porous materials, and has the advantages of simple operation and customization. Ge et al. employed acid etching to synthesize ultrafine defective RuO_2_ (UfD‐RuO_2_/CC) with an abundant pore structure and exposed active sites, which exhibited a lower overpotential (179 mV at 10 mA cm^−2^) compared with that of RuO_2_/CC (**Figure** **11**a–c).^[^ ^22b^ ^]^ The ultrathin and uniform Ru species was attained by dip‐coating, and acid etching was responsible for the increased number of Ru sites. Furthermore, the defects and vacancies in the ultrasmall RuO_2_ NPs were responsible for the improved stability and enhanced intrinsic activity.

However, atomic etching on a surface yields dynamic result. Particularly, different etching conditions yield different results. Zhou et al. found that a more thorough surface evolution of Ru sites occurred via the in‐situ electrochemical etching of Zn‐doped RuO_2_ (E‐Zn–RuO_2_) than via acid etching (C‐Zn–RuO_2_).^[^ ^115^ ^]^ During electrochemical etching, pre‐oxidation and irreversible surface reconstruction occurred on E‐Zn–RuO_2_, forming a stable active surface with more defects (Figure 11d). The E‐Zn–RuO_2_ catalyst exhibited excellent OER performance with a low overpotential of 190 mV at 10 mA cm^−2^ and stability for 60 h at the same current density (Figure 11e,f); this was because Zn doping and electrochemical etching modified the fine structure around Ru.

In contrast to obtaining porous materials through postprocessing, the one‐step synthesis of porous materials with different material characteristics is challenging, and this has been investigated.^[^ ^153^ ^]^ Li et al. prepared W–Ir–B alloy ingots with rich IrW nanochannels by arc‐melting high‐purity elements (>99.9 wt%) under a Ti‐gettered Ar atmosphere.^[^ ^152^ ^]^ These alloy ingots were remelted at least five times and shaped into rods. The W–Ir–B alloy achieved a current density of 2 A cm^−2^ at an overpotential of 497 mV and showed remarkable stability for 120 h at 100 mA cm^−2^, which was attributed to the ≈200 nm‐deep active IrW nanochannels (Figure 11h). The morphology of the catalyst did not change after the chronopotentiometric test, which confirmed the stability of the catalyst (Figure 11g). Chong et al. reported that La‐ and Mn‐codoped porous cobalt spinel fibers (LMCF) exhibited excellent performance with a current density of 2 A cm^−2^ at 2.47 V (Nafion 115 membrane) or 4 A cm^−2^ at 3.0 V (Nafion 212 membrane) in PEMWE device, which attributed to the porous and low‐dimensional nanostructure derived from Co‐ZIF and electrospinning technology. In this work, the durability of Co‐spinel oxide was significantly enhanced by removing the electrochemically unattached oxide, limiting metal ion dissolution due to the lack of electro‐potential stabilization. The primary size control and element doping further improve the performance from the mass‐transfer and corrosion‐resistance surface. The LSVs of LMCF before and after 14 000 voltage cycles in 0.1 m HClO_4_ are almost consistent, which demonstrated that the Co‐spinel oxide after element doping still maintains good stability under fluctuating voltage shocks. By doping large and stable atoms to form strain, vacancy and acid resistance on the surface of the catalyst, it provides guidance for the acid OER in acid unstable non platinum group materials.

MOFs are typical porous materials; however, they are typically difficult to stabilize in acidic media and thus cannot be used for the acidic OER. Hence, the design and synthesis of high‐performance MOFs catalysts for the acidic OER is challenging.^[^ ^154^ ^]^ Gao et al. developed a bipyridyl Th–MOF‐supported semirigid single‐site Co catalyst (CoCl_2_@Th–BPYDC), which exhibited a higher acidic OER activity than commercial IrO_2_, in addition to long‐term stability.^[^ ^155^ ^]^ This was attributed to the robust and rigid framework and strong interactions between the single metallic center and bipyridine N.

Consequently, compared with bulk catalysts, porous structures are beneficial for the diffusion of electrolytes, mass transfer, and high‐current‐density acidic OER.^[^ ^142^, ^152^ ^]^ Furthermore, the tailored porous structure exhibits significant potential for commercial PEMWEs. The highly accessible exposed surface area provides numerous active sites and enables easy access for reactants, as well as efficient removal of products. In PEMWEs, this porous structure facilitates gas diffusion from the catalyst's surface, reducing the likelihood of gas bubbles blocking active sites and thereby enhancing overall electrolyzer efficiency. The porous structure aids in managing water in the electrolyzer by facilitating the easy transport of water molecules to the membrane for hydration, a crucial process for proton conduction. Moreover, the porous structure contributes to the mechanical stability of the catalyst layer in PEMWEs, thereby enhancing the durability and longevity of these devices.

### 4.3 Low‐Dimensional Materials

Apart from the core–shell structure stabilizing the active center, other nanostructures play a crucial role in catalysis. Porous materials can address the physical stability problems of materials, enabling low‐dimensional materials to be better utilized. Low‐dimensional nanoscale materials such as nanowires,^[^ ^156^ ^]^ nanotubes, nanorods,^[^ ^15b^ ^]^ and NSs^[^ ^71^, ^73^, ^157^ ^]^ are being extensively used for the acidic OER. These low‐dimensional catalysts provide large specific areas and exposed active sites, which enhance the intrinsic activity and lifetime of the catalysts.

**Figure 12:**
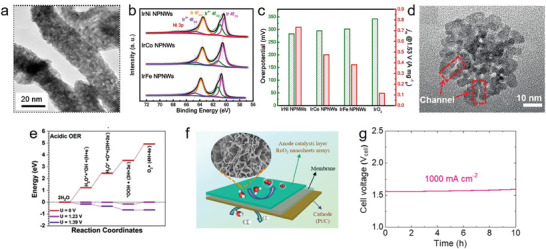
a) TEM images of the IrNi sample. b) XPS images of Ir 4f for IrM NPNWs. (M = Ni, Co, Fe). c) Bar graph showing overpotential (η) to drive 10 mA cm^−2^ and mass activity (*j*_m_) at 1.53 V vs RHE of the catalysts. a–c) Reproduced with permission.^[^ ^156^ ^]^ Copyright 2019, ACS Publications. d) High‐magnification TEM image of RuCu NSs. e) Reaction pathway of acidic OER on RuCu NSs. d,e) Reproduced with permission.^[^ ^73^ ^]^ Copyright 2019, Wiley‐VCH. f) Schematic of the membrane‐electrode assembly with RuO_2_‐NS/CF as the anode. g) Long‐term stability of RuO_2_‐NS/CF as an anode at a large current density of 1000 mA cm^−2^ in PEMWE. f,g) Reproduced with permission.^[^ ^157^ ^]^ Copyright 2021, Elsevier.

For example, Ir_0.1_Ta_0.9_O_2.45_ NPs exhibited excellent catalytic performance for the acidic OER because of their ultrasmall size (<2 nm), effective Ir dispersion on the surface, and atomic configuration suitable for the OER.^[^ ^158^ ^]^ Similarly, Liu et al. reported that sub‐2 nm IrO_2_/Ir nanoclusters effectively utilized the advantages of the active species.^[^ ^66b^ ^]^ Moreover, different IrM (M = Ni, Co, Fe) catalysts with low Ir contents and abundant nanopores were fabricated via a eutectic‐directed self‐templating strategy.^[^ ^156^ ^]^ The IrM catalysts exhibited a network structure owing to the entanglement of porous nanowires (**Figure** **12**a). The gap between the performance of the IrM catalysts was determined by the different transition metal species, and IrNi nanowires were found to be optimal (Figure 12c). The chemical states of the IrM catalysts were characterized (Figure 12b), and Ir^0^ and Ir^4+^ appeared in IrM, indicating that the fine structure of IrM was altered. Moreover, the negative shift confirmed that the electron transfer from the transition metal M to the Ir atoms modified the electronic structure of Ir and prevented its overoxidation. RuTe_2_ porous nanorods with an amorphous structure were also reported to present the advantages of 1D porous structures for acidic OER.^[^ ^15b^ ^]^ IrO_x_QD/GDY exhibited similar properties.^[^ ^159^ ^]^ The high exposure of the active sites of IrO_x_QD/GDY, rapid charge transfer, effective mass transfer, and ion diffusion, and facile gas release is related to the uneven charge distribution on the surface of GDY and the strong interactions between GDY and QDs.

Yao et al. reported that channel‐rich RuCu NSs exhibited excellent water‐splitting performance (Figure 12d).^[^ ^73^ ^]^ The final formation of O_2_ with an energy barrier of 0.164 eV was regarded as the RDS. The acidic OER occurred smoothly when the applied potential reached 1.39 V (Figure 12e). Furthermore, two‐dimensional NS catalysts were found to expose additional surface atoms and exhibit ultra‐high catalytic effects owing to the existence of defects at the edge.^[^ ^2^, ^67^ ^]^ Huang et al. developed a porous and defective RuO_2_ NSs with hierarchical structure aligned on carbon fibers (CF) (RuO_2_–NS/CF) (Figure 12f).^[^ ^157^ ^]^ The RuO_2_–NS/CF catalyst demonstrated excellent catalytic performance with a low overpotential of 212 mV at 10 mA cm^−2^ and high stability at a high current density (100 mA cm^−2^ for 50 h). The mass activity of RuO_2_–NS/CF was 60 times higher than that of commercial RuO_2_ at an overpotential of 300 mV because of its unique structure. The porous, two‐dimensional, and hierarchical structure of RuO_2_−NS/CF displayed a considerably larger electrochemically active surface area, increased number of exposed atoms, and active defective edges, which resulted in a high mass transfer capacity. Moreover, the RuO_2_–NS/CF was operated as an anode in PEMWE, and provided a current density of 1000 mA cm^−2^ with considerable stability (Figure 12g).

In summary, low‐dimensional catalysts typically exhibit enhanced performance owing to their distinctive structure, because additional active centers are exposed and new defective sites are constructed. These porous low‐dimensional catalysts are optimal candidates for high‐current‐density OER in acidic media, which is an advantage that bulk materials cannot easily achieve. In PEMWEs, the low‐dimensional materials, such as nanoparticles, nanowires, or nanosheets, have high surface area‐to‐volume ratios. This high surface area can facilitate more active sites for reactions and improve mass transport, leading to enhanced catalytic activity. Further specific modifications to the highly exposed surface‐active sites of low‐dimensional materials can significantly enhance catalytic activity and/or durability. Moreover, these modifications can improve compatibility with support structures, effectively addressing the performance and composition challenges of PEMWEs.

### 4.4 Self‐Supported Electrocatalysts

Self‐supported catalysts can be more easily scaled up for commercial electrolyzer applications compared to catalysts that require separate support material, due to their faster mass transfer, binder‐free nature, higher active site exposure, and difficult to fall off.^[^ ^160^ ^]^ Moreover, integrating the catalyst and support into a single structure can simplify electrode fabrication processes, potentially leading to more efficient manufacturing. Zhou et al. reported that Ru/TiO_x_ exhibited superior performance with an overpotential of 174 mV@ 10 mA cm^−2^ and 900 h long‐term stability at the same current density.^[^ ^11^ ^]^ In this work, the Ru nanoparticles (≈4 nm) had a high dispersion on the TiO_x_ nanorods, and then the catalyst‐supporter binding strength was stronger with spontaneous redox between Ru^3+^ and TiO_x_ after hydrothermal. Furthermore, this work confirms that the valence of Ru active center is significantly stabilized by the strong catalyst‐supporter interaction to hinder the overoxidation of Ru, thus enhancing the durability of catalyst for acidic OER. Deng and co‐workers also reported similar work. The CoO_x_/RuO_x_‐CC showed excellent performance with ultralow overpotential of 180 mV@ 10 mA cm^−2^ and stable operation in PEMWE for 100 h at 100 mA cm^−2^.^[^ ^161^ ^]^ The satisfactory performance is attributed to the reduced leaching and the inhibitory overoxidation of Ru by electron‐donated CoO_x_. This work proved that the strong catalyst‐support interaction is beneficial to the charge redistribution of Ru centers and weakens the covalence of Ru‐O bonds, which optimizes the binding strength of O‐intermediates and reduces the reaction barrier. Qiao et al. also reported that the reduced binding strength between HOO* and Ir sites and superior activity from higher Ir valence are attributed to the electronic interaction of Ir and Ta_2_O_5_.^[^ ^162^ ^]^

In addition to optimizing the electronic structure of the active center through heterostructure to achieve great performance, the self‐supported catalysts also expose a larger specific surface area by constructing rich pore/channel structures to accelerate the mass transfer effect and degassing efficiency, which is consistent with the challenges of PEMWEs. Recently, more studies have been conducted on dense nanowire arrays, abundant nanochannels, superfine nanofibers, etc., to meet the challenges of high current density, fast mass transfer, and long‐term stability of acidic OER.^[^ ^82^, ^152^, ^163^ ^]^ For example, Li et al. reported that in situ‐formed ∼200 nm deep IrW nanochannels are beneficial to the gas release and as a supporter stabilize the Ir oxide species to further enhance the activity.^[^ ^152^ ^]^ Zhang et al. developed a Ru‐based electrocatalyst with high aspect ratio morphologies to demonstrate that large exposure of Ru center significantly enhances activity and that close contact between the Ru center and supporter reduces interfacial charge transport resistance for efficient acidic OER.^[^ ^163b^ ^]^

Besides, the production of self‐supported catalysts can be scalable and cost‐effective, especially for continuous or large‐scale catalytic processes. Self‐supported catalysts have the potential to reduce overall system costs by eliminating the need for separate support materials, simplifying manufacturing processes, and potentially reducing catalyst loading requirements. Furthermore, self‐supported catalysts can be engineered to be more durable and resistant to degradation under the harsh operating conditions of water electrolysis, leading to longer lifetimes and reduced maintenance costs.^[^ ^164^ ^]^

While there are still challenges to address, such as optimizing the performance and durability of self‐supported catalysts under real‐world operating conditions, ongoing research and development efforts are focused on overcoming these challenges and unlocking the full potential of self‐supported catalysts for commercial PEMWEs (**Table** **3**).

**Table 3:** Typical catalysts of Structure Engineering for acidic OER.

Structure Engineering	Catalysts	Overpotential [mV] at 10 mA cm^−2^	Stability	Reference
Core–shell	RuNi_2_@G‐250	227	24 h@ 10 mA cm^−2^	Cui et al.^[^ ^144^ ^]^
	Si–RuO_x_@C	220	100 h@ 10 mA cm^−2^	Liu et al.^[^ ^34^ ^]^
	Co_3_O_4_@C/CP	370	86.8 h@ 100 mA cm^−2^	Yang et al.^[^ ^148^ ^]^
	40‐Co_3_O_4_@C/GPO	360±4	>40 h@ 10 mA cm^−2^	Yu et al.^[^ ^149^ ^]^
	FTO@Co_3_O_4_ NPs	511	21.5 h@ 10 mA cm^−2^	Yeh et al.^[^ ^150^ ^]^
	Ru–RuO_2_/GDY	163	75 h@ 10 mA cm^−2^	Chen et al.^[^ ^142a^ ^]^
	Ir–Co_3_O_4_@Co_3_O_4_	257	8 h@ 15 mA cm^−2^	Tran et al.^[^ ^146a^ ^]^
	Ru@RuO_2_	191	20 h@ 5 mA cm^−2^	Wen et al.^[^ ^165^ ^]^
	Ru@Ir–O	238	40 h@ 10 mA cm^−2^	Zhang et al.^[^ ^142d^ ^]^
Porous	UfD‐RuO_2_/CC	179	20 h@ 10 mA cm^−2^	Ge et al.^[^ ^22b^ ^]^
	E‐Zn–RuO_2_	190	60 h@ 10 mA cm^−2^	Zhou et al.^[^ ^115^ ^]^
	CoCl_2_@Th–BPYDC	388	25 h@ 1.681 V vs. RHE	Gao et al.^[^ ^155^ ^]^
	Ir p‐NHs	243	/	Bao et al.^[^ ^142b^ ^]^
	LMCF	353±30	353 h@ 10 mA cm^−2^	Chong et al.^[^ ^17a^ ^]^
	SrIr_2_O_6_	303	300 h@ 10 mA cm^−2^	Wang et al.^[^ ^166^ ^]^
	Ir_3_Ni NCs	282	12 h@ 3 mA cm^−2^	Ding et al.^[^ ^167^ ^]^
	Se‐RuO_2_ aerogel	166	48 h@ 10 mA cm^−2^	Han et al.^[^ ^168^ ^]^
	CdRu_2_IrO_x_	189	1500 h@ 10 mA cm^−2^	Liu et al.^[^ ^169^ ^]^
	Ru‐UiO‐67‐bpydc	200	115 h@ 10 mA cm^−2^	Yao et al.^[^ ^170^ ^]^
Low‐dimensional	RuO_2_–NS/CF	212	50 h@ 100 mA cm^−2^	Huang et al.^[^ ^157^ ^]^
	IrO_x_/GDY	236	30 h@ 10 mA cm^−2^	Wang et al.^[^ ^159^ ^]^
	Ir‐IrO_x_/C nanosheets	198	8 h@ 10 mA cm^−2^	Zu et al.^[^ ^76^ ^]^
	Ir‐Cu/C NSs	237	24 h@ 5 mA cm^−2^	Mahmood et al.^[^ ^171^ ^]^
	nC–Bi_2_Te_3_	160	22 h@ 10 mA cm^−2^	Arbab et al.^[^ ^172^ ^]^
	UF‐Ir/IrO_x_	299	200 h@ 10 mA cm^−2^	Chen et al.^[^ ^173^ ^]^
	RuCoO_x_	200	100 h@ 10 mA cm^−2^	Zhu et al.^[^ ^174^ ^]^
Self‐supported	Ru/TiO_x_	174	900 h@ 10 mA cm^−2^	Zhou et al.^[^ ^11^ ^]^
	CoO_x_/RuO_x_‐CC	180	60 h@ 10 mA cm^−2^	Deng et al.^[^ ^161^ ^]^
	Ir/Ta_2_O_5_	218	200 h@ 100 mA cm^−2^	Qiao et al.^[^ ^162^ ^]^
	W–Ir–B alloy	∼497(@ 2 A cm^−2^)	120 h@ 100 mA cm^−2^	Li et al.^[^ ^152^ ^]^
	py‐RuO_2_: Zn	173	1000 h@ 10 mA cm^−2^	Zhang et al.^[^ ^163b^ ^]^

## 5 Conclusion and Prospects

Hydrogen is one of the most environmentally friendly energy sources worldwide. EWS has emerged as a convenient and promising technology for hydrogen production and has attracted considerable interest. Although PEMWE presents the most efficient option for hydrogen production, its widespread adoption is limited primarily because of anode materials. PEM electrolysis is inhibited by the sluggish kinetics of water oxidation at the anode. Generally, anode materials cannot withstand high oxidation potentials and corrosive acidic environments; this highlights the importance of anode‐material stability in the acidic OER. Ir‐based materials serve as benchmark catalysts for the acidic OER; however, the scarcity of Ir in the Earth's crust hinders its widespread application. Consequently, low‐Ir‐content and more robust Ir‐based catalysts must be developed for future applications. Additionally, Ru‐based materials, non‐precious metals, and metal‐free catalysts must be examined to advance this field. Defect, phase, and structure engineering are pivotal for guiding the design and synthesis of efficient acidic OER catalysts. Despite these advancements, the EWS mechanism remains unclear, necessitating further investigation to promote the development of PEMs. Accordingly, we propose the following strategies:

1) Maximum utilization of Ir/Ru‐based materials: The rapid development of hydrogen energy is a significant challenge for EWS system, especially PEMWEs. The development of high‐efficiency “low‐Ir/Ru content” catalysts for PEMWEs anode electrode, including nanocrystals, clusters, and atomic dispersions, has become an economical solution to this problem. ADCs have considerable research potential because of their 100% atomic utilization; however, their loading capacity is typically closely related to activity. Therefore, increasing the number of active sites can improve the acidic OER performance. This is also a challenge for the acid corrosion resistance and high oxidation potential resistance of the supporter, which should be carefully chosen based on safety, economy, durability and mechanical strength.
2) Development of non‐noble metal and metal‐free acidic OER catalysts: Due to the limited availability of noble metals, there is a pressing need to develop alternative non‐noble metal and metal‐free catalysts for acidic OER. Many non‐noble metal oxides, such as Co_3_O_4_ and MnO_2_, exhibit excellent activity, but their stability remains inadequate. To address this, the electronic structures of Co‐ and Mn‐based oxides need to be regulated to enhance their stability during operation. Additionally, carbon‐based materials emerge as promising candidates for the acidic OER. Functionalized carbon‐based materials often display exceptional catalytic performance, warranting further investigation.
3) Improved stability of catalysts: Stability is a crucial parameter for acidic OER catalysts because of their high oxidation potential and acid corrosion, which hinder electrocatalytic processes. Methods to ensure the stability of catalysts during acidic OER operation must be studied. Unexpected events resulting from the interactions between the support and active centers may also require investigation.
4) Accelerated lifetime test systems (ALTs): ALTs help to identify potential failure modes and degradation mechanisms, thereby enabling the development of more robust and reliable catalysts, even PEMWE systems. In electrochemical water splitting, Chronoamperometry (CA), chronopotentiometry (CP), and cyclic voltammetry (CV) are three traditional methods to obtain the lifetime data of catalysts. CA and CP are the most commonly used methods, which have been examined rigorously, and their validity has been confirmed. However, considering the future application scenario of PEMWEs, real‐time coupling with fluctuating renewable energy sources, CV is obviously the most suitable ALT testing method. Meanwhile, CV testing can simulate the effects of long‐term operation in a relatively short period of time, accelerate the evaluation of catalyst durability and stability, and help improve development efficiency and reduce costs.
5) Development of in‐situ technologies to elucidate the water splitting mechanism: a) We have not completely understood the exact mechanism and dynamic process of water splitting thus far. A standard approach is currently unavailable. Generally, different catalysts exhibit different mechanisms. Although the current in‐situ technology can clarify the reaction mechanism of the acidic OER, it cannot completely account for all the intermediates. Therefore, more advanced in‐situ technology and instruments are required to further study the mechanism of the acidic OER. b) Furthermore, more advanced, and finer in‐situ detection technologies should be used in the observation of the catalyst's surface evolution. For example, the participation of crystal water in the reaction requires in‐situ XRD to monitor the evolution of the crystal structure, or the formation of new protective layer species on the catalyst's surface needs to include but not be limited to in‐situ/non‐in‐situ detection methods to identify. Some works demonstrate the participation of crystal water with isotope tracing; however, the further detection of catalyst structure evolution is more meaningful and beneficial to the deep understanding of the water‐splitting mechanism.
6) Several challenges exist for PEMWEs: The cost of materials, especially the Pt‐ and Ir‐based catalysts, membranes, and bipolar plates, contributes to the high capital cost of PEMWE systems. The degradation of pivotal component (catalysts and membranes) is the primary concern in future commercial PEMWEs, which direct depend on cost‐effectiveness for widespread commercial adoption. Moreover, the PEM selectively allow protons to pass while preventing hydrogen gas crossover, which is a significant challenge for safety. Finally, some engineering challenges also should be solved, such as efficient water management to maintain optimal hydration levels in the membrane and prevent flooding or drying out, the work efficiency and durability of PEMWEs with varying electrical loads and intermittent renewable energy sources, etc.

## Conflict of Interest

The authors declare no conflict of interest.
